# Comparative transcriptome analysis to identify candidate genes involved in 2-methoxy-1,4-naphthoquinone (MNQ) biosynthesis in *Impatiens balsamina* L.

**DOI:** 10.1038/s41598-020-72997-2

**Published:** 2020-09-30

**Authors:** Lian Chee Foong, Jian Yi Chai, Anthony Siong Hock Ho, Brandon Pei Hui Yeo, Yang Mooi Lim, Sheh May Tam

**Affiliations:** 1 School of Biosciences, Faculty of Health and Medical Sciences, Taylor’s University, Jalan Taylors, 47500 Subang Jaya, Selangor Malaysia; 2 Faculty of Applied Sciences, UCSI University, Jalan Puncak Menara Gading, UCSI Heights, 56000 Cheras, Wilayah Persekutuan Kuala Lumpur Malaysia; 3 Fairview International School, Lot 4178, Jalan 1/27d, Seksyen 6 Wangsa Maju, 53300 Kuala Lumpur, Wilayah Persekutuan Kuala Lumpur Malaysia; 4 Department of Pre-Clinical Sciences, Faculty of Medicine and Health Sciences, Universiti Tunku Abdul Rahman, Lot PT 21144, Jalan Sungai Long, Bandar Sungai Long, 43000 Kajang, Selangor Malaysia

**Keywords:** Gene expression, Genetics, Genomics, Transcriptomics

## Abstract

*Impatiens balsamina* L. is a tropical ornamental and traditional medicinal herb rich in natural compounds, especially 2-methoxy-1,4-naphthoquinone (MNQ) which is a bioactive compound with tested anticancer activities. Characterization of key genes involved in the shikimate and 1,4-dihydroxy-2-naphthoate (DHNA) pathways responsible for MNQ biosynthesis and their expression profiles in *I. balsamina* will facilitate adoption of genetic/metabolic engineering or synthetic biology approaches to further increase production for pre-commercialization. In this study, HPLC analysis showed that MNQ was present in significantly higher quantities in the capsule pericarps throughout three developmental stages (early-, mature- and postbreaker stages) whilst its immediate precursor, 2-hydroxy-1,4-naphthoquinone (lawsone) was mainly detected in mature leaves. Transcriptomes of *I. balsamina* derived from leaf, flower, and three capsule developmental stages were generated, totalling 59.643 Gb of raw reads that were assembled into 94,659 unigenes (595,828 transcripts). A total of 73.96% of unigenes were functionally annotated against seven public databases and 50,786 differentially expressed genes (DEGs) were identified. Expression profiles of 20 selected genes from four major secondary metabolism pathways were studied and validated using qRT-PCR method. Majority of the DHNA pathway genes were found to be significantly upregulated in early stage capsule compared to flower and leaf, suggesting tissue-specific synthesis of MNQ. Correlation analysis identified 11 candidate unigenes related to three enzymes (NADH-quinone oxidoreductase, UDP-glycosyltransferases and S-adenosylmethionine-dependent O-methyltransferase) important in the final steps of MNQ biosynthesis based on genes expression profiles consistent with MNQ content. This study provides the first molecular insight into the dynamics of MNQ biosynthesis and accumulation across different tissues of *I. balsamina* and serves as a valuable resource to facilitate further manipulation to increase production of MNQ.

## Introduction

Increased focus on bio-based economy (or bioeconomy) has meant a renewed drive in the plant biotechnology sector to produce high-value bio-ingredients for various downstream applications. Bioeconomy emphasizes on sustainable (‘green’) production of renewable biological resources and their conversion into value-added products such as food, feed, chemicals, energy and healthcare and wellness products. Plants secondary metabolites have always been a source of biomaterial for many medicinal and industrial applications^1–6^. The rose balsam *Impatiens balsamina* L. (Balsaminaceae) is an annual tropical herbaceous plant which in whole or part has been used as traditional medicine (remedies) in Asian countries such as Japan, Korea, China, Taiwan, Thailand, Malaysia and India to treat various ailments^7–14^. Previous pharmacological studies reported promising antipruritic, anti-dermatitic, antihistaminic, antimicrobial (antibacterial and antifungal), analgesic, antioxidant, anti-inflammatory, anti-rheumatic, anti-anaphylactic, antitumor and anticancer activities from the testings of various *I. balsamina* extracts, often correlated with higher presence of natural compounds including quinones, anthocyanins, glycosides, alkaloids, saponins, flavonoids/flavanols, phenolics as well as terpenoids^10,14–18^.

From various *I. balsamina* extracts, the compound 2-methoxy-1,4-naphthoquinone (MNQ) is of notable interest. MNQ has shown anticancer and anti-metastatic properties in-vitro against HepG2 hepatocarcinoma cells^19^, MDA-MB-231 breast cancer cells^20,21^, A549 lung adenocarcinoma cells^22^, and MKN45 gastric adenocarcinoma cells^23^. A previous study on MNQ distribution in different parts of *I. balsamina* reported that capsules (pods) had the greatest amount of MNQ (8–150 folds difference) compared to flowers, roots, stems, leaves and seeds^10^. Other studies quantified naphthoquinones (e.g. MNQ, its precursor 2-hydroxy-1,4-naphthoquinone (lawsone) and methylene-3,3′-bilawsone) in specific tissues such as leaves^19,24–26^, stems^27^, roots^28^, and pericarps^8^. Comparison of results from these studies generally indicated that MNQ (and lawsone) accumulated at differing degrees in parts of *I. balsamina*.

Naphthoquinones such as MNQ and lawsone comprise a subclass of quinones, structurally related to naphthalene and characterized by the substitution of the naphthalene skeleton at position C1 and C4 (1,4-naphthoquinones) or C1 and C2 (1,2-naphthoquinones)^29^. Naphthoquinones (K vitamins, phylloquinone, menaquinone) and other related quinones such as benzoquinones (ubiquinone and plastoquinone) and anthraquinones are generally synthesized via the polyketide-, shikimate- and isoprenoid pathways^30–32^. Other naturally occurring 1,4 naphthoquinones known in higher plants include plumbagin (5-hydroxy-2-methyl-1,4-naphthoquinone)^33^; lapachol (2-hydroxy-3-(3-methyl-2-butenyl)-1,4-naphthoquinone)^34^; juglone (5-hydroxy-1,4-naphthoquinone)^35,36^ and shikonins (5,8-dihydroxy-2-((1R)-1-hydroxy-4-methyl-3-penten-1-yl)-1,4-naphthalenedione)^37^.

**Figure 1:**
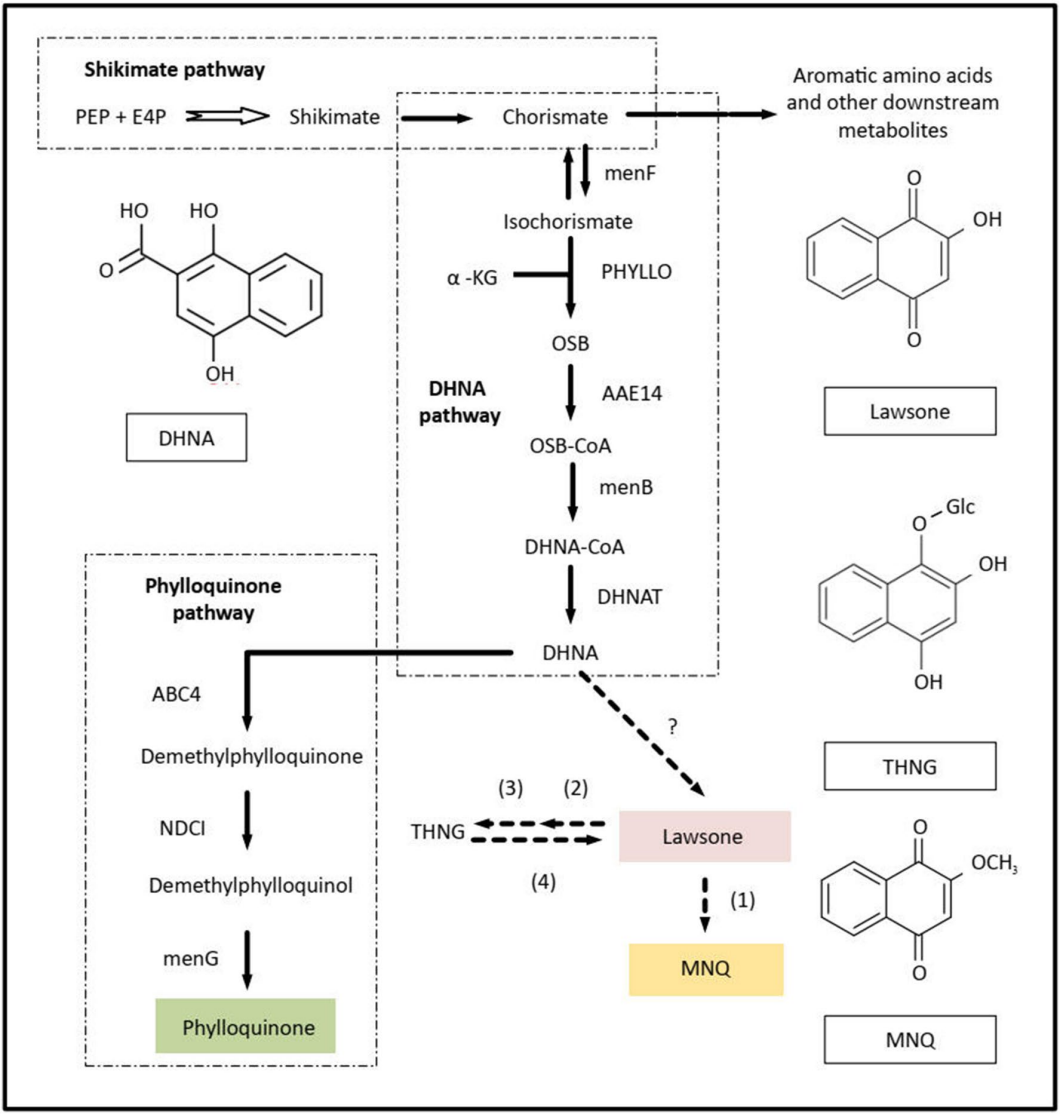
Biosynthesis of MNQ based on known and postulated connections between the key intermediate DHNA and its downstream 1,4-naphthoquinones; phylloquinone, lawsone and MNQ. D-erythrose 4-phosphate (E4P) and phosphoenolpyruvate (PEP) are the starting substrate for the biosynthesis of shikimate. In the 1,4-dihydroxy-2-naphthoate (DHNA) pathway, α-ketoglutarate (α-KG) is added and isochorismate synthase (PHYLLO) converts isochorismate into o-succinylbenzoate (OSB) leading to formation of DHNA, which serves as a starting substrate for the biosynthesis of phylloquinone, lawsone and MNQ. Dotted-line arrows indicate postulated enzymes responsible for the later steps in the biosynthesis of lawsone and MNQ (Widhalm and Rhodes^32^): (1) S-adenosylmethionine-dependent o-methyltransferase (SAM dependent o-MT); (2) oxidoreductase; (3) glycosyltransferase; (4) unknown β-glucosidase. *AAE14* acyl-activating enzyme 14 (EC:6.2.1.26), *ABC4* DHNA phytyl transferase, *DHNAT* 1,4-dihydroxy-2-naphthoyl-CoA thioesterase (EC:3.1.2.28), *menB* naphthoate synthase (EC:4.1.3.36), *menF* menaquinone-specific isochorismate synthase (EC:5.4.4.2), *menG* demethylphylloquinone methyltransferase, *NDC1* NAD(P)H dehydrogenase C1, *THNG* 1,2,3-trihydroxynaphthalene-1-O-glucoside. DHNA and phylloquinone pathways are adapted from KEGG^51,52^. Chemical structures were produced using ChemDraw Jr 18.1 (https://chemdrawdirect.perkinelmer.cloud/js/sample/index.html).

Previous genetic and biochemical studies had found that the 1,4-naphthalenoid ring was derived from shikimate^38,39^ and O-succinylbenzoate (OSB)^40^, thus implicating the shikimate- and OSB pathways, also known as the 1,4-dihydroxy-2-naphthoate (DHNA) pathway in the production of MNQ^32^. The shikimate pathway consists of six core enzymatic reactions resulting in the synthesis of chorismate, which is the starting compound for the subsequent seven reactions in the DHNA pathway. The catalytic activity of a trifunctional enzyme, PHYLLO converts chorismate to OSB, where it is sequentially catalysed to form OSB-CoA, then 1,4-dihydroxy-2-naphthoate-CoA (DHNA-CoA) and finally hydrolysed into DHNA by the enzymes acyl-activating enzyme 14 (AAE14), naphthoate synthase and DHNA thioesterase (DHNAT), respectively^41^. DHNA is a key precursor used in the biosynthesis of phylloquinone (2-methyl-3-phytyl-1,4-naphtho-quinone or vitamin K_1_) in plants, in addition to other specialized 1,4-naphthoquinones such as lawsone^39,42^, juglone^36^, anthraquinones^43^, and lapachol^34^. In *I. balsamina*, only phylloquinone and lawsone are directly derived from DHNA (Fig. 1), with lawsone being the precursor of MNQ^32^. Three enzymatic reactions are required to convert DHNA into phylloquinone and this pathway has been fully characterized due to the latter’s importance as an electron carrier in photosystem I (PSI) during photosynthesis^44^. However, the enzymes for specialized 1,4-naphthoquinones biosynthesis downstream of DHNA have not been identified, including that for MNQ biosynthesis. What is currently reported is that lawsone is formed via oxidative decarboxylation of DHNA by an unknown enzyme, and an enzyme with S-adenosylmethionine-dependent O-methyltransferase activity was proposed to convert lawsone to MNQ^32,45^. In terms of transport and storage stability, the functions of an oxidoreductase to reduce lawsone followed by a glycosyltransferase to produce a glucosylated form of reduced lawsone (1,2,4-trihydroxynaphthalene-1-O-glucoside, THNG) were also postulated, as THNG had been isolated in *I. glandulifera*, and most probably in *I. parviflora* and *I. balsamina*^46^. Currently, none of the genes involved in MNQ biosynthesis pathways has been characterized for *I. balsamina*, although many studies exist on its bioactivities, total content, and different extraction and purification methods.

In this study, quantification of MNQ and lawsone in different tissues of *I. balsamina* were performed using High-Performance Liquid Chromatography (HPLC) analysis; and the transcriptomes of leaf, flower, and capsules in three stages of development (early-, mature- and postbreaker-stages) of *I. balsamina* were generated using Illumina HiSeq4000 paired-end sequencing technology and analysed. HPLC results ascertained that comparatively higher amounts of MNQ are distributed in pericarps of *I. balsamina*, in contrast to lawsone which was mainly present in leaves*.* Key findings from comparative analysis of the transcriptomes include successful characterization of all the genes of the shikimate and DHNA pathways for *I. balsamina;* and correlation analysis of differential gene expression patterns and spatial distribution of MNQ suggests de novo synthesis of MNQ in the capsules of *I. balsamina*, and allowed identification of 11 candidate unigenes encoding three enzyme classes proposed to be involved in the final steps of MNQ biosynthesis in *I. balsamina.* Overall, the transcriptomes and results obtained from this study provide a basis for the further analysis of the biosynthetic pathways and serve as a resource for further research towards increased production of natural MNQ.

## Material and methods

### Plant material

**Figure 2:**
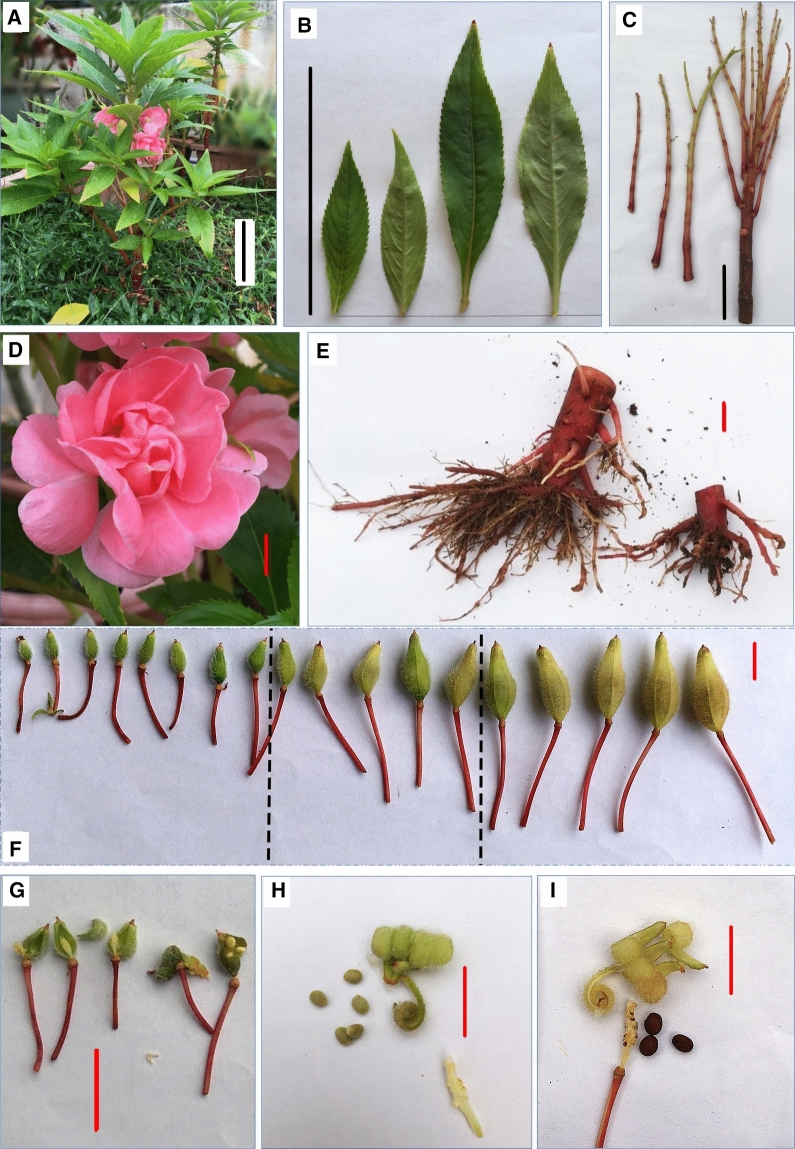
Morphology of different tissue parts of the rose balsam *Impatiens balsamina* used in this study. (**A**) Whole plant, (**B**) Young- (left) and mature (right) leaves, (**C**) Stems, (**D**) Flower, (**E**) Roots, (**F**) Capsules at different developmental stages, i.e. early-, mature-, and postbreaker stages (from left to right), (**G**) Early stage pericarp and seeds, (**H**) Mature stage pericarp and seeds, and (**I**) Postbreaker stage pericarp and seeds. Scale bar: black line = 10 cm, red line = 1 cm.

Cultivated plants of the pink, multi-petal form of *I. balsamina* were obtained from a local nursery (Kajang, Selangor, Malaysia) and then continually seed propagated in a home garden setting (externally in an open condition). For HPLC quantification, *I. balsamina* plants were grown from 1st of July to 8th of September 2017, in 1-L growth bags using a mix of black garden soil and clay (2:1). For this period, the average high and low temperatures recorded were 32 °C and 23 °C respectively, photoperiod of 12:12 light: dark cycle, and mean rainfall of 161.67 mm (Kajang, Malaysia Meteorological Department). The plants were watered twice daily (morning and evening) except when it rained, and standard fertilizer (N:P:K 5:3:2) was applied once every 2 weeks. Tissues were harvested from healthy, 10 weeks old plants on 9th September 2017 and pooled following these criteria (Fig. 2): mature leaves (between 50 and 100 mm in length, 3rd or 4th branch from shoot apex), young leaves (≤ 50 mm length, 1st or 2nd branch from shoot apex), stems, roots, flowers (open/blossomed), pericarps and seeds from capsules at three developmental stages i.e. early- (within 3–13 days after anthesis, length ≤ 12 mm, pericarp green, seeds white), mature- (within 15–25 days after anthesis, length 15–18 mm, pericarp green, seeds brown), and postbreaker (within 26–31 days after anthesis, length ≥ 20 mm, pericarp yellow-green, seeds dark brown). For each tissue, a minimum of three to six replicates were extracted for HPLC quantification. For total RNA extraction, five tissue types were harvested from 10 weeks old plants according to the criteria stated above. Mature leaf, flower, and capsules at three developmental stages (early-, mature- and postbreaker stage were collected on 9th September 2017 and immediately placed into RNAlater solution (Ambion, Austin, TX, USA) prior to total RNA extraction.

### HPLC quantification of MNQ and lawsone content

Freshly collected plant tissues were dried separately using silica gel at room temperature, ground to fine powder, and stored in falcon tubes in the dark at 25 °C prior to extraction. Solvent extraction was performed for each sample using ethyl acetate (1:100 ratio; 1 g 100 mL^−1^) for 7 days (solvent was replaced every 3 days) at 25 °C under continuous shaking at 120 rpm. The extracts were then filtered, solvent evaporated using a rotary evaporator (30 °C, 90 hPa, 120 rpm), re-dissolved with 6 mL ethyl acetate and left till the solvent evaporated to dryness in the dark. Dried residues were reconstituted with methanol, adjusted to concentrations of 1000–2500 ppm, and filtered through a 0.45 µm membrane filter. HPLC analysis was carried out on a Shimadzu 20A series HPLC system (Shimadzu, Kyoto, Japan) with a Brownlee Analytical C18 column at 25 °C. Each run was set at 20 min with gradient elution as follows: 15 min at composition 95:5 (acetonitrile: water), 4 min at 5:95, and 2 min at 95:5. Flow rate was set as 1 mL min^−1^ with sample injection volume of 20 µL and detection by UV at wavelength 266 nm. Standards of lawsone and MNQ (Sigma Aldrich, St. Louis, MO, USA) prepared in methanol at five concentrations (20, 40, 60, 80 and 100 ppm). Standards of lawsone and MNQ (100 ppm) were also used to spike samples during HPLC analysis for peak validation. Standard curves were constructed from the analysis of the reference standards (five different concentrations, minimum of three replicates, two separate HPLC runs) and plotting peak area against the concentration of each reference standard. The regression equation and coefficient of determination (R^2^) were calculated, and linearity was expressed in terms of correlation coefficient (r). Quantification of compounds from different samples was done by comparing sample peak areas against the standard curves. All statistical analysis was performed using SPSS version 23 (SPSS Incorporation, Chicago, IL, USA) and the data were subjected to one-way analysis of variance (ANOVA) to determine differences between groups. Tukey’s post hoc test or Games-Howell (assumption of variance not assumed) test was performed for inter-group comparison and ρ-value ≤ 0.05 was considered significant.

### Total RNA extraction, cDNA library construction, and transcriptome sequencing

Total RNAs were extracted following an optimized protocol described by^47^. Values of A_260/280_ and A_260/230,_ RNA integrity number (RIN) and 28S/18S ribosomal RNA ratio of the samples were measured using a NanoDrop 1000 spectrophotometer (NanoDrop Technologies, Wilmington, DE, USA), and Agilent 2100 Bioanalyser (Agilent RNA 6000 Nano Kit; Agilent Technologies, Santa Clara, CA, USA). For each tissue type, two samples (replicates) with RIN ≥ 6.5 and OD260/280 and OD260/230 values ≥ 1.8 were used for transcriptome sequencing. cDNA libraries were constructed using the TruSeq RNA Library Prep Kit v2 (Illumina, San Diego, CA, USA) following the manufacturer’s protocol, using the oligo(dT) method for mRNA isolation. The mRNAs were fragmented to 300–500 bp before cDNA synthesis. Purified cDNA fragments were then added with single nucleotide A (adenine), ligated to adapters and PCR enriched. Paired-end sequencing was performed using HiSeq 4000 (Illumina, San Diego, CA, USA) that generated a minimum of five Gb of clean reads per sample by BGI Tech Solution (Hong Kong) CO., LIMITED (Hong Kong, China).

### Transcriptome data processing and de novo assembly

Raw data were processed to eliminate low-quality reads, Illumina adapter sequences, and reads with high content of unknown bases (N). Resulting clean reads after filtering were de novo assembled using Trinity program^48^, and TGICL^49^ was used to cluster transcripts, eliminate redundancy and obtain unigenes. TransDecoder software^50^ was used to predict coding regions (open reading frames, ORF) of the unigenes (default parameters, minimum of 100 amino acid sequence). The longest ORFs were then subjected to BLAST analysis against SwissProt and Hmmscan databases to obtain Pfam protein homology sequence for the prediction of coding DNA sequences (CDS).

### Functional annotation and classification of unigenes

Functional annotation of the assembled unigenes were performed by sequence comparisons via BLASTN, BLASTX or Diamond at default parameters against seven public databases, namely the NCBI protein database (NR), NCBI nucleotide database (NT), Eukaryotic Orthologous Groups (KOG), Kyoto Encyclopedia of Genes and Genomes (KEGG)^51,52^, Gene Ontology (GO), SwissProt and InterPro. GO- and InterPro annotations were achieved using Blast2Go^53^ and InterProScan5^54^, respectively.

### Unigene expression and differentially expressed genes (DEGs) analysis

#### Unigene expression

All clean reads were mapped to unigenes using Bowtie2^55^, and gene expression level calculated with the Expectation–Maximization (RSEM) software package^56^. Expression abundance of the unigenes was represented as the number of fragments per kilobase of exon model per million mapped reads (FPKM).

#### DEGs analysis

DEGs were identified between pairwise comparison of five tissue types of leaf (L), flower (F), early- (E), mature- (M) and postbreaker (P) stage capsules, totalling ten comparisons using DEseq2^57^ following the criteria of minimum fold change ≥  ± 2.00 and adjusted ρ-value ≤ 0.05, with expression ratios expressed as FPKM values. Distribution of DEGs detected for each pair of comparison is summarized and visualized through heatmaps. GO classification and functional enrichment analyses of the DEGs were performed using R program (with ‘phyper’ function) to determine the distribution of the DEGs of each comparison tissue group in the three primary ontology classes of molecular function, cellular component and biological process. The DEGs identified were also subjected to KEGG pathway enrichment analyses. The GO and KEGG pathway terms were considered significantly enriched with a corrected Ρ-value ≤ 0.05.

### 1,4-Dihydroxy-2-naphthoate (DHNA) biosynthesis pathway gene expression analysis

Hierarchical clustering analysis was performed based on the log-transformed FPKM values using R studio^58^ with hclust function to analyse DEGs identified between different tissue groups related to annotated genes involved in the 1,4-dihydroxy-2-naphthoate (DHNA) biosynthesis pathway in *I. balsamina* as well as candidate genes postulated to function in the last steps of MNQ biosynthesis (downstream of the DHNA intermediate). Correlation analysis of the candidate genes was conducted using nonparametric Spearman R method with the default two-tailed ρ-value and 95% confident interval.

### Validation of DEGs with quantitative real-time PCR (qRT-PCR) analysis

To verify expression data shown by the transcriptomes, qRT-PCR was performed on 20 selected genes from the terpenoids backbone- (mevalonate, MVA and 2-C-methyl-d-erythritol 4-phosphate, MEP), shikimate- and DHNA pathways. The *I. balsamina* total RNA samples used in the qRT-PCR assays were the same batch as those used for the transcriptome sequencing. Primers for each of the gene were designed using Primer 3 tool (https://primer3.ut.ee/) following the criteria of GC% of 45–55% and melting temperature of 55–60 °C (Supplementary Table S1). First strand cDNA synthesis was performed using Tetro cDNA Synthesis Kit (Bioline, London, UK) with oligo (dT)_18_ primer according to the manufacturer’s instructions. Sample cDNAs were diluted to a final concentration of 100 ng/µL. qRT-PCR was performed in Eppendorf RealTime PCR Cap Strips (Eppendorf, Hamburg, Germany) using SensiFAST SYBR No-Rox Kit (Bioline, London, UK). qRT-PCR reactions were performed in triplicates for each gene and tissue part, in a total 20 µL reaction containing 300 ng template cDNA, 1X SensiFAST No-Rox mix, 400 nM forward and reverse primers, and adequate nuclease- and RNase-free water using a MasterCycler EP Gradient Thermal Cycler (Eppendorf, Hamburg, Germany). Cycling conditions involved an initial denaturation of 95 °C/2 min, followed by 40 cycles of 95 °C/5 s, primer-specific annealing temperature at 60 °C/10 s and extension at 72 °C/10 s. The melting curve for each amplicon was performed from 60° to 95 °C to verify primer specificity. Aldolase, elongation factor 1-alpha (EF1a) and ubiquitin-conjugated enzyme (UCE) genes served as internal reference genes and were used to normalise the gene expression data. Relative expression level of target genes was calculated using the 2^−∆∆CT^ method^59,60^, with the following formula:

$$\begin{aligned} 2^{{ - \Delta \Delta {\text{C}}_{{\text{T}}} }} & = \left[ {\left( {{\text{C}}_{{\text{T}}} \;{\text{gene}}\;{\text{of}}\;{\text{interest}} - {\text{C}}_{{\text{T}}} \;{\text{internal}}\;{\text{control}}} \right){\text{sample}}\;{\text{A}}} \right. \\ & \quad \left. { - \left( {{\text{C}}_{{\text{T}}} \;{\text{gene}}\;{\text{of}}\;{\text{interest}} - {\text{C}}_{{\text{T}}} \;{\text{internal}}\;{\text{control}}} \right)\;{\text{sample}}\;{\text{B}}} \right]~ \\ \end{aligned}$$

A linear regression model was used to correlate the log-transformed relative quantification value of the genes from qRT-PCR results with the respective log-transformed relative gene expression values in the transcriptome data.

## Results

### Lawsone and MNQ contents in *Impatiens balsamina*

**Figure 3:**
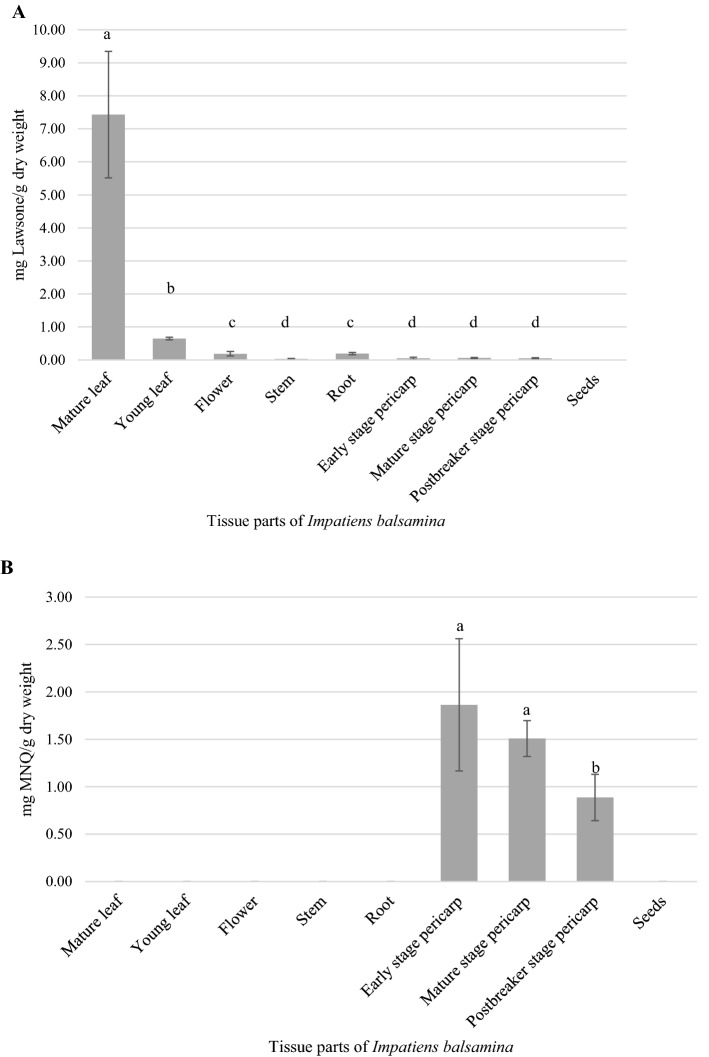
Quantified amounts of (**A**) 2-hydroxy-1,4-naphthoquinone (lawsone) and (**B**) 2-methoxy-1,4-naphthoquinone (MNQ) extracted from different tissue parts of the pink multi-petal *Impatiens balsamina*. Average total contents (mg g^−1^) were calculated from multiple samples (with three to six biological replicates) analyzed from two HPLC runs. Content of lawsone is the sum of the three lawsone tautomeric HPLC peaks (at the retention time of 1.844, 2.051, 2.284 min). ^a–d^Labels with different letters indicate a significant difference (Games Howell test, p < 0.05) in the average contents between samples.

To investigate the relationship between MNQ content and gene expression, contents of lawsone and MNQ in different tissues of *I. balsamina* were determined by HPLC. As seen in Fig. 3, results confirmed that lawsone and MNQ accumulated at significantly different quantities in distinct tissues of *I. balsamina* (p ≤ 0.05). Quantification of lawsone based on the standard curve generated showed that the total average lawsone content was 8.662 mg g^−1^ dry weight. Mature leaves, at an average of 7.431 ± 1.915 mg g^−1^ dry weight yielded the highest amount of lawsone, a difference of ~ 9-folds (p ≤ 0.05) compared to young leaves (0.650 ± 0.039 mg g^−1^), with significantly lesser amounts of lawsone recorded in roots (0.195 ± 0.034 mg g^−1^), flowers (0.191 ± 0.067 mg g^−1^), stems (0.033 ± 0.013 mg g^−1^), early- (0.051 ± 0.035 mg g^−1^), mature- (0.060 ± 0.017 mg g^−1^) and postbreaker stage pericarps (0.051 ± 0.020 mg g^−1^). Lawsone was not detected in the seed samples. Two to three retention peaks were observed in mature leaves and standards during the lawsone HPLC analysis, validated as lawsone from similar retention times (RT), spectra profiles, and spiking using standard solution in multiple HPLC runs (Supplementary Figs. S1A, S2A; Supplementary Tables S2, S3). These two/three peaks observed correspond to the three known tautomeric forms of lawsone (1,4-naphthoquinone, 1,2-naphthoquinone and 1,2,4-naphthotrione)^61,62^, and suggests concentration may influence tautomer formation. It was reported that different concentrations and temperature have effects on favouring either the enol or keto form of 7-hydroxyquinolines tautomer in equilibrium^63^, thus further research is needed to determine affecting factors of lawsone tautomers. As shown in Fig. 3, MNQ was only detected in the pericarps (all three stages) of *I. balsamina.* Based on the standard curve generated, a total average content of 4.259 mg g^−1^ dry weight was calculated, i.e. the average content of MNQ quantified were 1.864 ± 0.697 mg g^−1^, 1.508 ± 0.189 mg g^−1^ and 0.887 ± 0.244 mg g^−1^ dry weight for the early-, mature- and postbreaker stage pericarps respectively (Supplementary Figs. S1B, S2B; Supplementary Tables S2, S3).

### Transcriptome sequencing of *Impatiens balsamina* and de novo assembly

Paired-end transcriptome sequencing generated 59.643 Gb of total raw reads for the five sets of transcriptomes comprising of leaf, flower, and three capsule developmental stages (early-, mature- and postbreaker stages) of *I. balsamina*, with two biological replicates for each tissue*.* The raw transcriptome data have been deposited in NCBI GenBank with Sequence Read Archive (SRA) accession PRJNA526137. Summary of the sequencing output for the ten transcriptomes from *I. balsamina* is shown in Table 1. After filtering of low-quality reads, adaptor trimmed and unknown (N) base reads, 55.194 Gb of clean reads were de novo assembled into 595,828 transcripts with average length and N_50_ size of 1057 bp and 1646 bp respectively. After eliminating redundancy, a total of 94,659 unigenes were obtained, with an average length of 1222 bp and N_50_ value of 1925. GC percentages for the ten transcriptomes ranged from 42.48 to 43.36% (average 42.94%; Table 1) and 42.49 to 43.40% (average 42.94%; Supplementary Table S4) for the assembled transcripts and unigenes respectively.

**Table 1:** Sequencing output for the ten transcriptome libraries from leaf (L), flower (F), and three developmental stages of capsules (early- (E), mature- (M) and postbreaker (P) stages) of *Impatiens balsamina.*

Sample^a^	Total raw reads (Mb)	Total clean reads (Mb)	Total clean nucleotides (Gb)	N50 of transcripts	GC (%)
L1	58.49	54.74	5.47	1545	42.84
L2	59.16	55.02	5.50	1525	42.81
F1	58.35	54.77	5.48	1554	43.30
F2	61.26	56.44	5.64	1552	43.36
E1	58.64	54.76	5.48	1598	43.24
E2	59.69	54.96	5.50	1576	43.22
M1	60.74	56.00	5.60	1524	42.89
M2	59.84	54.26	5.43	1522	42.82
P1	59.04	55.03	5.50	1573	42.48
P2	61.22	55.96	5.60	1596	42.48

^a^Each tissue has two biological sample sequencing outputs.

### Functional annotation of *Impatiens balsamina* unigenes

Overall, 70,008 unigenes (73.96%) of *I. balsamina* were successfully annotated, with the highest percentage of annotation (68.78%, 65,104 unigenes) achieved against the NR database. Other annotation results against SwissProt, Interpro, KEGG, KOG, NT and GO are summarized in Supplementary Table S5. It was shown that 26.04% of the unigenes did not possess significant similarity to sequences of other species. The species distribution map based on NR annotation revealed high similarities with *Vitis vinifera* (15.63%), *Sesamum indicum* (6.63%), *Coffee canephora* (5.41%), *Theobroma cacao* (4.3%), and *Nicotiana sylvestris* (3.36%) (Supplementary Fig. S3). TransDecoder predicted 75,204 ORFs from the *I. balsamina* unigenes corresponding to N_50_ of 1317, GC content of 44.68% and maximum and minimum lengths of 16,089 and 297 respectively. Based on GO annotation, the unigenes were classified into 55 subcategories of biological process, cellular component, or molecular function (Supplementary Fig. S4), while 55,114 *I. balsamina* unigenes (58.22%) were annotated by KOG database and classified into 25 categories of functional class (Supplementary Fig. S5). KEGG annotation resulted in the assignment of *I. balsamina* unigenes into a total of 138 pathways (Supplementary Fig. S6).

### Analysis of unigenes expression

Based on the assembly results, all clean reads for each sample were mapped back to the unigenes and the FPKM value of each unigene were thus calculated and used to measure gene expression level. A total of 92,026 unigenes were found to be expressed in one or more tissue types, with postbreaker stage capsule having the highest number of expressed unigenes (average of 64.64% from 94,659 total unigenes), followed by leaf (average of 62.87%), early stage capsule (average of 62.72%), mature stage capsule (average 57.94%), and flower (average of 52.73%). The total number of expressed unigenes in each tissue is presented in Supplementary Table S6.

### Differentially expressed genes (DEGs) analysis between leaf, flower, early-, mature- and postbreaker stage capsules of *Impatiens balsamina*

**Figure 4:**
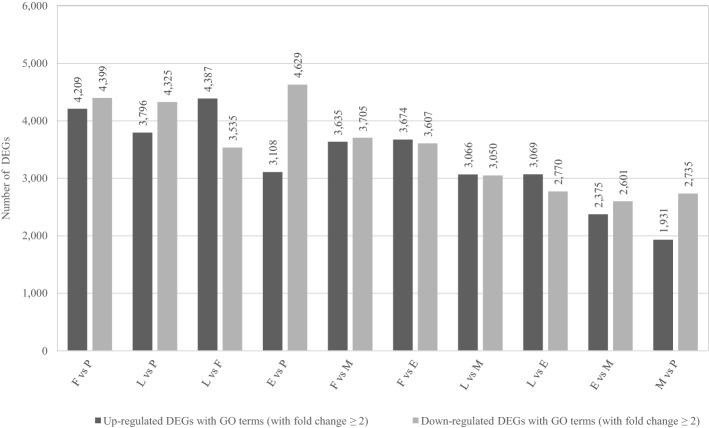
Overview of differentially expressed genes (DEGs) among the leaf (L), flower (F), and early- (E), mature- (M) and postbreaker- (P) stage capsules in *Impatiens balsamina*. Numbers of up-regulated and down-regulated DEGs of each pairwise comparison from analysis of all types of unigenes (clusters and singletons) from leaf, flower and the three stages of capsules.

A total of 50,786 DEGs out of 92,026 unigenes expressed (55.19%) were detected using DESeq2 (fold change ≥  ± 2.00 and adjusted ρ-value ≤ 0.05) from individual pairs of comparison between the five tissue types. Among the ten pairwise comparisons between leaf (L), flower (F), early- (E), mature- (M) and postbreaker- (P) stage capsules, the highest and lowest DEGs observed were between L vs. P with 23,916- (11,895 up- and 12,021 down-regulated) and M vs. P with 12,877- (5849 up- and 7028 down-regulated) DEGs respectively (Fig. 4). Hierarchical clustering of the DEGs for all ten comparisons are displayed in Supplementary Fig. S7.

### Gene ontology (GO) enrichment and KEGG pathway assignments of DEGs

**Figure 5:**
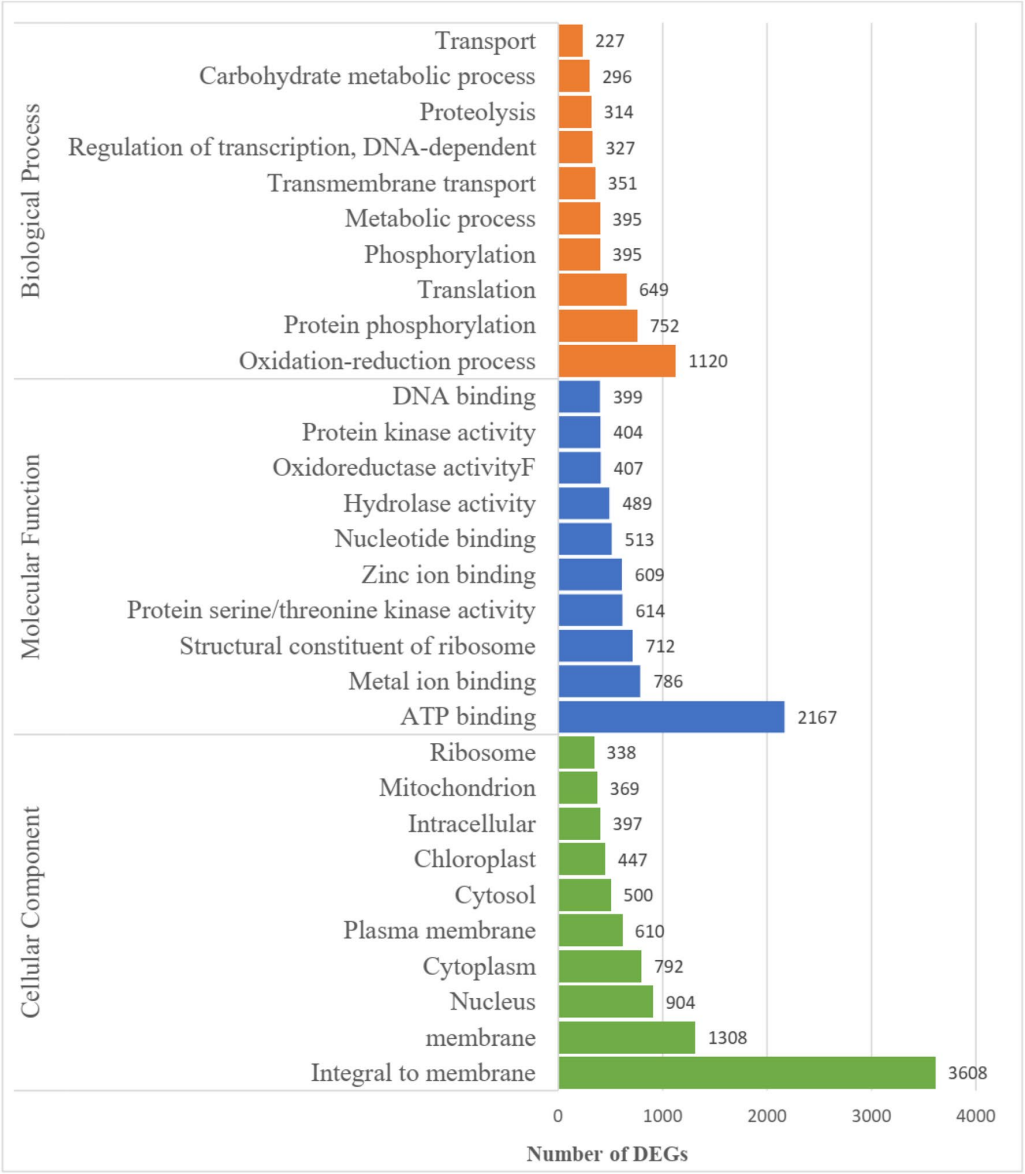
Overview of enriched GO terms for differentially expressed genes (DEGs) of *Impatiens balsamina* among five different tissues.

Results of GO classification and functional enrichment analysis of 17,151 DEGs (33.77% of 50,786 DEGs) are presented in Supplementary Table S7. Overall, the three most significantly enriched terms (ranked by corrected ρ-value) for the DEGs classified in the categories of cellular component were ‘integral to membrane’ (7.10%), ‘membrane’ (2.58%), and ‘nucleus’ (1.78%); the terms ‘ATP binding’ (4.27%), ‘metal ion binding’ (786 DEGs), ‘structural constituent of ribosome’ (1.40%) for molecular function; and ‘oxidation–reduction process’ (2.21%), ‘protein phosphorylation’ (1.48%), ‘translation’ (1.28%) were most enriched in biological process. Under ‘metabolic process’, the subcategories of ‘secondary metabolic process’ (GO:0019748), ‘secondary metabolite biosynthesis process’ (GO:0044550), and ‘biosynthetic process’ (GO:0009058) contained 0.01%, 0.05%, and 0.14%, respectively (Fig. 5).

KEGG pathway enrichment results allowed for better understanding of the functions of the DEGs, with 66.71% DEGs (out of 50,786 DEGs) mapped to 174 KEGG pathways (Supplementary Table S8). Overall, the DEGs were mostly classified in ‘Metabolism’ (57.08% of total KEGG enriched DEGs), followed by ‘Human diseases’ (52.01%), ‘Organismal Systems’ (31.60%), ‘Environmental Information Processing’ (19.70%), ‘Genetic Information Processing’ (13.72%), and ‘Cellular Processes’ (12.01%). DEGs which were assigned with KEGG ID but not mapped to any pathway accounted for 1086 unigenes (2.14%). With regards to the relevant pathway terms involved in secondary metabolisms, ‘Biosynthesis of other secondary metabolites’ contained 1.86% DEGs, whereby 0.78%- and 0.30% DEGs were classified under the subcategories of ‘Phenylpropanoid biosynthesis’ and ‘Flavonoid biosynthesis’ respectively. Other pathways included ‘Metabolism of terpenoids and polyketides’ with 1.30% DEGs: subcategories ‘Terpenoid backbone biosynthesis’ (0.32%), ‘Carotenoid biosynthesis’ (0.27%) and ‘Sesquiterpenoid and triterpenoid biosynthesis’ (0.23%); ‘Metabolism of cofactors and vitamins’ contained 1.80% with subcategories of ‘Ubiquinone and other terpenoid-quinone biosynthesis’ 0.16% DEGs; Under the level 2 term of ‘Amino acid metabolism’ (3.72%), ‘Phenylalanine, tyrosine and tryptophan biosynthesis’ contained 0.26% DEGs.

### Biosynthesis of MNQ in *Impatiens balsamina* as revealed by DEGs analysis of the shikimate- and DHNA pathways

Functional annotation successfully identified all the genes (enzymes) for *I. balsamina* involved in the shikimate- (Supplementary Table S9) and DHNA pathways. Expression data of 27 *I. balsamina* unigenes involved in the DHNA pathway were mapped onto the pathway. The end-product of this pathway, i.e. DHNA is a key precursor for producing MNQ. HPLC quantification showed that MNQ contents were singly higher in capsules, compared to leaves and flowers of *I. balsamina,* thus allowing exploration of MNQ biosynthesis by comparing expression data of the DHNA pathway genes in different tissues respectively.

As seen in Table 2A, most of the unigenes corresponding to each DHNA pathway gene were expressed one-to-five-fold higher in early stage capsule compared to flower: *menF* (log_2_FC = 3.477), majority of the unigenes encoding *PHYLLO* (log_2_FC range − 2.029 to 4.750), *AAE14* (log_2_FC range 3.363–3.693), *menB* (log_2_FC range 1.542–5.478) and *DHNAT* (log_2_FC range 1.191–4.094), supporting the biosynthesis of MNQ in the capsule of *I. balsamina*. In addition, lawsone and MNQ are both synthesized from the same core DHNA pathway, with lawsone (detected mainly in leaves) being the immediate precursor of MNQ (abundant in capsules). The observation that relative expression levels of most DHNA pathway genes were significantly up-regulated in early stage capsule even when compared to leaf (which produces lawsone) suggests that MNQ is de novo synthesized in early stage capsule. In early stage capsule, four of the five DHNA pathway genes were significantly higher than in leaf: *menF* (log2FC = 4.220), most unigenes encoding *PHYLLO* (log2FC range 2.932–3.726), *AAE14* (log2FC range 3.009–4.319) and *menB* (log2FC range 3.668–5.068). Only *DHNAT*, which functions to convert DHNA-CoA to DHNA, did not show significant difference i.e. DHNAT was highly expressed in both leaf and early stage capsule (Table 2A).

**Table 2:** Relative expression profile of genes from the (A) 1,4-dihydroxy-2-naphthoate (DHNA) and (B) phylloquinone biosynthesis pathways in *Impatiens balsamina* between early stage capsule, leaf and flower.

Gene	Unigene ID	Log_2_ fold change (up-/down-regulation)^a^	
		Early stage capsule vs. flower	Early stage capsule vs. leaf
**(A) DHNA biosynthesis pathway**			
*menF*	CL4612.Contig2_All	3.477 (Up)	4.220 (Up)
*PHYLLO*	CL4092.Contig3_All	2.750 (Up)	3.041 (Up)
	CL4092.Contig2_All	4.750 (Up)	2.932 (Up)
	Unigene24037_All	1.143 (Up)	3.172 (Up)
	CL4092.Contig1_All	− 0.087	3.726 (Up)
	Unigene23373_All	0.250	0.287
	Unigene23381_All	− 2.029 (Down)	− 0.232
	Unigene21821_All	3.519 (Up)	0.723
*AAE14*	CL1366.Contig2_All	3.449 (Up)	4.319 (Up)
	CL1366.Contig1_All	3.363 (Up)	4.828 (Up)
	CL1366.Contig3_All	3.693 (Up)	3.009 (Up)
*menB*	CL643.Contig2_All	5.478 (Up)	3.957 (Up)
	CL643.Contig1_All	4.146 (Up)	3.917 (Up)
	Unigene8203_All	4.086 (Up)	4.066 (Up)
	Unigene17346_All	3.168 (Up)	4.648 (Up)
	Unigene17345_All	2.619 (Up)	4.327 (Up)
	Unigene17344_All	1.542 (Up)	5.068 (Up)
	Unigene17347_All	4.288 (Up)	3.668 (Up)
	Unigene5523_All	0.875	− 0.102
*DHNAT*	CL6893.Contig7_All	− 0.026	− 0.341
	CL6893.Contig3_All	0.028	− 0.288
	CL6893.Contig5_All	1.191(Up)	0.529
	CL6893.Contig6_All	2.402	0.097
	CL6893.Contig8_All	2.102	1.385
	CL6893.Contig2_All	1.966	− 0.293
	CL6893.Contig4_All	− 0.316	0.526
	CL6893.Contig1_All	4.094 (Up)	0.080
**(B) Phylloquinone biosynthesis pathway**			
ABC4	CL9909.Contig1_All	2.274 (Up)	− 0.782
	CL9909.Contig2_All	1.501 (Up)	− 0.739
	CL9909.Contig3_All	2.132 (Up)	− 0.727
	Unigene36201_All	1.647	2.081
NDC1	CL2069.Contig1_All	− 0.122	0.124
	CL2069.Contig2_All	0.738	− 0.845
	CL2069.Contig3_All	2.041	0.835
	CL2069.Contig4_All	− 0.807	− 0.959
	CL2069.Contig5_All	− 2.219	− 4.675 (Down)
	CL2069.Contig7_All	0.528	− 1.381 (Down)
	CL2069.Contig8_All	− 0.117	− 0.549
	CL2069.Contig9_All	0.536	0.787
	CL10396.Contig2_All	0.227	0.582
menG	Unigene17701_All	0.811	0.992

Log_2_ fold-change values were obtained based on normalised DESeq2 counts in the respective DEG pairwise comparisons.

^a^Significant up- or down-regulation is determined based on Fold Change ≥ 2.00 and Adjusted P-value ≤ 0.05. See Fig. 1 for the abbreviation of gene names.

DHNA is a branch point intermediate (key precursor) for the biosynthesis of phylloquinone, and early stage capsule may possess photosynthetic activity because it is green (Fig. 2). Phylloquinone, due to its PSI function are synthesized and accumulated in green and photosynthetic parts (e.g. leaves), in contrast to other non-photosynthetic parts of the plant^41^. Formed in three-steps starting with the conversion of DHNA to demethylnaphthoquinone via the phytylation process of DHNA phytyl transferase (ABC4)^64,65^; then reduction to demethylphylloquinol involving demethylnaphthoquinone oxidoreductase [or NAD(P)H dehydrogenase C1, NDC1], phylloquinone is finally formed by demethylphylloquinone methyltransferase^66,67^. As shown in Table 2B, differential expression analysis revealed *ABC4* was significantly up-regulated in early stage capsule (significant log_2_FC of E vs. F ranged from 1.501 to 2.274) compared to flower, suggesting that early stage capsule is likely to possess photosynthesis activity attributed to active expression of phylloquinone-related genes. However, no significant difference was detected in the expressions of *ABC4* and *2-phytyl-1,4-beta-naphthoquinone methyltransferase* (*menG*) between leaf and early stage capsule in *I. balsamina*. In fact, *NAD(P)H dehydrogenase C1* (*NDC1*) was significantly down-regulated in early stage capsule (significant log_2_FC of E vs. L ranged from − 4.675 to − 1.381) compared to leaf (Table 2B). This provide more convincing evidence that early stage capsule has higher expressions of DHNA pathway genes to synthesize DHNA to cater for MNQ production and not solely for phylloquinone.

Correlating well with the amount of MNQ quantified, expressions of all five DHNA pathway genes then underwent significant down-regulation in the mature- and postbreaker stage capsules compared to early stage capsule (Supplementary Table S10): *menF* (log2FC in mature- and postbreaker stage capsules vs. early stage capsule were − 2.044 and − 6.315, respectively), *PHYLLO* (max. log2FC of − 3.006 and − 3.647, respectively), *AAE14* (max. log2FC of − 2.182 and − 10.732, respectively), *menB* (max. log2FC of − 2.475 and − 9.620, respectively), and *DHNAT* (max log2FC of − 2.376 and − 1.658, respectively).

### Identification of candidate genes involved in the late steps of MNQ biosynthesis in *Impatiens balsamina* based on correlation analysis

To produce MNQ, lawsone is first synthesised via oxidative decarboxylation of DHNA by an unknown enzyme. For the subsequent conversion of lawsone to MNQ, the activity of a ‘S-adenosylmethionine-dependent O-methyltransferase’ (SAM-dependent O-MT) was postulated^45^. From annotation results of the *I. balsamina* transcriptomes, a total of 104 unigenes with ‘SAM-dependent O-MT activity’ were found and clustered in a heatmap (Supplementary Fig. S8). For identification of SAM-dependent O-MT candidates, correlation results found six unigenes showing significant positive correlation of gene expression with MNQ content. Upon examination of their DEG values, three candidate unigenes (CL2491.Contig8_All, *p* = 0.0087; CL2491.Contig17_All, *p* = 0.0074; CL2491.Contig3_All, p = 0.0023) were further shortlisted, with expressions that showed consistent, significant upregulation (four to seven folds) in ‘early stage capsule vs. flower’ and ‘early stage capsule vs. leaf’ respectively (Table 3).

**Table 3:** Expression profile of shortlisted putative S-adenosylmethionine-dependent O-methyltransferase genes correlated to MNQ content in distinct tissues of *Impatiens balsamina.*

Unigene ID	Correlation analysis	log_2_ fold change (up-/down-regulation)		SwissProt annotation results
	Correlate to MNQ content	Early stage capsule vs. leaf	Early stage capsule vs. flower	
CL2491.Contig8_All	0.808**	6.905 (Up)	6.190 (Up)	Q9M571 Phosphoethanolamine *N*-methyltransferase
CL2491.Contig17_All	0.818**	6.217 (Up)	4.090 (Up)	Q9M571 Phosphoethanolamine *N*-methyltransferase
CL2491.Contig3_All	0.886**	5.720 (Up)	4.992 (Up)	Q9M571 Phosphoethanolamine *N*-methyltransferase

Correlation analysis was performed using Spearman correlation method. Significant values are marked with asterisk mark, which ** refers to p-value ≤ 0.01. Significant up- or down-regulation is determined based on normalized DEG analysis, with Fold Change ≥ 2.00 and Adjusted P-value ≤ 0.05.

In addition, it is also known that a reduced, glycosylated form of lawsone (THNG) exists, generated using an oxidoreductase that uses NADH or NADPH as electron donors as well as a glycosyltransferase^32^. The *I. balsamina* transcriptomes contained 82 unigenes with the description ‘oxidoreductase activity, acting on NAD(P)H’, and 122 unigenes with ‘UDP glycosyltransferases’. Results of the correlation analysis identified a total of three- and five candidate unigenes encoding ‘NADH-quinone oxidoreductase’ and ‘UDP glycosyltransferases’ respectively, both based on expression patterns showing significant positive relationships to lawsone content (p ≤ 0.01) (Table 4). In this case, it was assumed that lawsone produced would be transformed into THNG for stability, solubility, transport and sequestration, as well as to physiologically inactivate the compound in the plant^68^. Nucleotide sequences of these candidate unigenes are provided in Supplementary Table S11.

**Table 4:** Expression profile of shortlisted putative oxidoreductase and UDP-glycosyltransferase genes correlated to lawsone content in distinct tissue of *Impatiens balsamina*.

Unigene ID	Correlation analysis	log_2_ fold change (up-/down-regulation)		SwissProt annotation results
	Correlate to lawsone content	Leaf vs. flower	Leaf vs. early stage capsule	
**(A) Oxidoreductase**				
Unigene20901_All	0.763*	3.059 (Up)	1.524 (Up)	Q49KU3 NAD(P)H-quinone oxidoreductase subunit 1, chloroplastic
CL7647.Contig1_All	0.711*	6.991 (Up)	1.852 (Up)	Q2HXL0 Respiratory burst oxidase homolog protein C
CL9700.Contig1_All	0.689*	6.766 (Up)	1.363 (Up)	NA
**(B) UDP-glycosyltransferase**				
CL1643.Contig5_All	0.985****	1.318 (Up)	1.945 (Up)	Q2V6K0 UDP-glucose flavonoid 3-O-glucosyltransferase 6
CL9133.Contig1_All	0.886**	4.365 (Up)	4.291 (Up)	O82627 Granule-bound starch synthase 1, chloroplastic/amyloplastic
CL2812.Contig11_All	0.886**	1.967 (Up)	1.383 (Up)	Q9ZQ95 UDP-glycosyltransferase 73C6
CL1844.Contig2_All	0.739*	5.418 (Up)	4.548 (Up)	O64733 UDP-glycosyltransferase 87A2
CL9133.Contig2_All	0.689*	4.701 (Up)	4.321 (Up)	O82627 Granule-bound starch synthase 1, chloroplastic/amyloplastic

Correlation analysis was performed using Spearman correlation method. Significant values are marked with asterisk mark, which * refers to p-value ≤ 0.05, ** refers to p-value ≤ 0.01, and **** refers to p-value ≤ 0.0001. Significant up- or down-regulation is determined based on normalized DEG analysis, with Fold Change ≥ 2.00 and Adjusted P-value ≤ 0.05. NA = not annotated to SwissProt database.

### Quantitative real-time PCR validation

**Figure 6:**
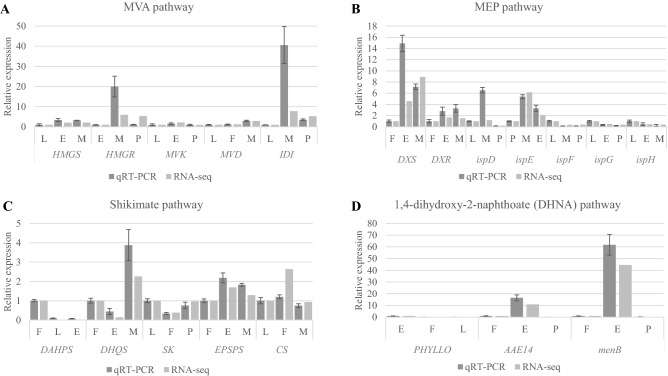
Comparison of differential expression results of 20 selected secondary metabolism genes in different tissues of *Impatiens balsamina* obtained by qRT-PCR and RNA-seq. Each qPCR result represents the mean of log2fold change values (± standard deviation) of three biological replicates obtained from leaf (L), flower (F), early- (E), mature- (M), and postbreaker (P) stage capsules using the ∆∆CT method (qRT-PCR column), in comparison with the respective expression level of the gene obtained from the *I. balsamina* transcriptome dataset (RNA-seq column). See Supplementary Table S1 for gene name abbreviation.

To validate the transcriptomes, qRT-PCR were performed for 20 selected genes from the MVA-, MEP-, shikimate- and DHNA pathways on combinations of three tissue types (Fig. 6; Supplementary Tables S12 and Table S13). Melting curve analysis performed by qRT-PCR after 40 cycles of amplification detected the presence of single peaks indicating the expected amplicons were amplified for each gene. Results of linear regression analysis indicated a relatively high correlation (R^2^ = 0.7962) of log-transformed gene expression (fold changes) between the normalized qRT-PCR and transcriptome datasets (Supplementary Fig. S9), suggesting the RNA-Seq data are reliable. However, six out of 20 selected genes were observed to show much higher DEGs in qRT-PCR compared to transcriptome results. Expression of *HMGR* (E vs. M) was 20.000-fold change as calculated in qRT-PCR but underestimated in the transcriptome (5.902-fold change from RNA-seq). Similar observations were noted for *IDI* (L vs. M; 40.564-fold change in qRT-PCR vs. 7.762-fold change in RNA-seq), *DXS* (F vs. E; 14.900- vs. 4.580-fold change), *ispD* (L vs. M; 6.550- vs. 1.202-fold change), *DHQS* (F vs. M; 3.876- vs. 2.257-fold change) and *menB* (F vs. E; 61.744- vs. 44.565-fold change).

## Discussion

Both MNQ and lawsone quantified using HPLC analysis were shown to be present in significantly different concentrations in distinct tissues of *I. balsamina.* The overall amount of lawsone isolated from *I. balsamina* (average of 0.866% w w^−1^ dry weight) was found to be comparable to the henna plant, *Lawsonia inermis* (1–1.4% w w^−1^)^69^. MNQ was detected only in the three pericarp stages of *I. balsamina* at a total average content of 4.259 mg g^−1^ dry weight, similar to a previous finding^10^ which also reported the highest amount of MNQ being isolated from the pericarps of *I. balsamina.* In our study, the lawsone content extracted was ~ two folds higher than MNQ, concurring with a previous study^70^ for *I. capensis, I. noli-tangere* and *I. parviflora*. Unlike MNQ which was only detected in the capsules, lawsone was found in various parts of *I. balsamina*, including leaf, flower, and root, consistent with a recent study by^71^ on *I. glandulifera*. MNQ and lawsone were detected in the leaves, fresh seed pod capsules, roots, and whole flowers of *I. glandulifera*. Natural variation in the distribution of lawsone and MNQ in different parts of *I. balsamina* may be explained by the physiological roles of these compounds such as plant defense (antimicrobial, natural insecticides), allelopathy and UV absorption^10,71–74^, contributing to the ecological success of *I. balsamina.*

In this study, the transcriptomes of leaf, flower, early-, mature- and postbreaker stage capsules of *I. balsamina* were generated and analysed. The transcriptome sequencing outputs and functional annotation results obtained (total of 94,659 unigenes obtained, 73.96% unigenes successfully annotated) are comparable to the recently reported transcriptome results of *I. walleriana* and *I. hawheri*^75^. In the *I. balsamina* transcriptome datasets, 26.04% of the unigenes did not possess significant similarity to sequences of other species, which is close to the percentage of ‘orphans’ or ‘taxonomically restricted genes’ (TRGs) in a given species^76^. TRGs are known as genes in a given species that do not have homologs in other species and postulated to account for 10–20% of genes in eukaryotic genomes^76,77^. These genes are likely to be related to the evolution of novelty and adaptive species-specific processes^78^. To confirm the robustness of the *I. balsamina* transcriptomes, qRT-PCR of 20 selected genes were performed and the analysed results (R^2^ = 0.80) indicate reliability of the transcriptome data.

The biosynthesis of MNQ involves two major pathways, namely the shikimate- and DHNA pathways. All the genes (enzymes) of both these pathways for *I. balsamina* were successfully identified from the transcriptomes. Differential expression of the DHNA pathway genes in five different tissues allowed the gaining of significant insights into the biosynthesis of MNQ in *I. balsamina*. DEG analysis revealed that majority of the genes involved in the DHNA pathway (up to synthesis of DHNA) were highly and significantly expressed in early stage capsule compared to flower and leaf, validating MNQ biosynthesis and further suggestive of de novo formation of DHNA in the capsule of *I. balsamina* leading to final production of MNQ. It was also observed that the highest expression of DHNA pathway genes occurred in early stage capsule, and were then down-regulated in mature- and postbreaker stage capsules. This suggests that the biosynthesis of DHNA is highly active in early stage of capsule, gradually declining in the later stages of capsule development, correlating well with MNQ content in the pericarps of the three stages of capsules.

DHNA is a compound potentially diverted into two different pathways for the biosynthesis of phylloquinone and MNQ. Phylloquinone is a primary metabolite important for its function as an electron carrier in photosystem I (PSI) during photosynthesis. It was observed that higher expression of *ABC4* occurred in early stage capsule compared to flower but no significant change compared to leaf, but *NDC1* was down-regulated in early stage capsule compared to leaf. These results are indicative of the presence of functionally active phylloquinone pathway genes in early stage capsule, which could be explained by the fact that developing fruits can be photosynthetically active^79^. Pericarps have some ability to perform photosynthesis that has been proposed to play a notable role in seed growth and development in tomato^80,81^, wheat and barley^82,83^, *Mercurialis annua* and other Euphorbiaceae^84^, and certain species of Brassicaceae^85^. Results of *NDC1* unigenes encoding the second enzyme of the phylloquinone pathway showing lower expressions combined with higher expressions of DHNA pathway genes in early stage capsule compared to leaf, serve to suggest that DHNA produced in early stage capsule is sufficient to support MNQ production in situ*,* branching off from the other DHNA downstream pathway i.e. phylloquinone biosynthesis.

From the correlation analyses of gene expression data and MNQ (and lawsone) content in different tissues of *I. balsamina*, a total of 11 unigenes were shortlisted from the transcriptomes that corresponded to the three enzyme classes proposed to catalyse the synthesis of MNQ (via lawsone) in capsules. According to Swissprot annotation, the three SAM-dependent O-MT shortlisted candidate unigenes mainly encode phosphoethanolamine *N*-methyltransferase, an enzyme that plays a key role in the synthesis of the metabolite phosphatidylcholine via a phospho-base methylation pathway in plants^86,87^. The additional candidate unigenes related to lawsone biosynthesis identified encoding NADH-quinone oxidoreductase are either respiratory burst oxidase homologs or Cytochrome P450s. Respiratory burst oxidase homologs are plant NADPH oxidase that plays key roles in cellular signalling network of reactive oxygen species and various processes such as plant development, hormonal and environmental stresses^88–92^. Cytochrome P450s, such as 71A1 is involved in the metabolism of compounds associated with the development of flavour in the fruit ripening process^93,94^, and 77A2 was found to involved in the flower bud development^95,96^. Candidate unigenes identified for the second putative enzyme of ‘UDP glycosyltransferases (UGT)’ corresponded to several glycosyltransferases (GTs), particularly UDP-glucose flavonoid 3-O-glucosyltransferase 6 (GT6), UGT73C6 and UGT87A2. UGTs catalyse glycosylation which is one of the final steps in producing secondary metabolites^68^. UGTs belong to the subfamily of GTs that play an important role in plant secondary metabolism^97,98^, known to participate in the regulation of hormones and biosynthesis of secondary metabolites such as indolyl-3-butyric acid, cytokinin^99–101^ flavonoids, phenylpropanoids, terpenoids, steroids^102^, and flavanol glycoside^103^, although functions of most UGTs are still unknown^97,98,104^. Using an approach combining quantitative HPLC and comparative transcriptome analysis, putative candidate genes involved in MNQ downstream pathway have been identified, especially the S-adenosyl-l-methionine-dependent methyltransferases will warrant further studies to functionally validate their respective roles in the biosynthesis of MNQ.

## Conclusions

In this study, de novo transcriptome sequencing and analyses of the leaf, flower and early-, mature- and postbreaker stage capsules allowed identification of all the annotated genes involved in the shikimate and DHNA pathways responsible for the production of MNQ in *I. balsamina*. Correlation between expression of shikimate- and DHNA pathway genes with MNQ pools, combined with knowledge of previous labeling experiments by^32,45,46^ suggest that MNQ biosynthesis branches off the phylloquinone pathway. Significant upregulation of most genes of the DHNA pathway in early stage capsule compared to flower and leaf suggests that MNQ is synthesized de novo in a tissue-specific manner in the capsule of *I. balsamina*. A total of 11 candidate unigenes corresponding to the enzyme families of S-adenosylmethionine O-methyltransferases, oxidoreductases, and UDP glycosyltransferases postulated to catalyse the final reaction of MNQ production as well as lawsone stability were identified based on their expression levels being significantly and positively correlated with MNQ- and lawsone content in different tissues of *I. balsamina*. Knowledge and better understanding of the genes involved in these biosynthesis pathways (and their expression patterns) now provide the required genomics resource for targeted manipulation of these pathways either via genetic engineering or synthetic biology.

## Supplementary information

Supplementary Information.

Supplementary Table S8.

Supplementary Table S9.

Supplementary Table S11.

## Data Availability

Raw sequence reads of the reported *Impatiens balsamina* transcriptomes are available at NCBI Sequence Read Archive under BioProject accession number PRJNA526137 (https://www.ncbi.nlm.nih.gov/bioproject/526137; Release date: 2020-04-10 or upon publication of this manuscript).
