# Supplementary material for: Comparative transcriptome analysis to identify candidate genes involved in 2-methoxy-1,4-naphthoquinone (MNQ) biosynthesis in Impatiens balsamina L
Source: Sci Rep. 2020 Sep 30;10:16123. doi: 10.1038/s41598-020-72997-2 (PMC7527972; doi:10.1038/s41598-020-72997-2)
Supplement: Supplementary file 1 — Supplementary Information. [file 41598_2020_72997_MOESM1_ESM.docx]

**Comparative transcriptome analysis and evaluation of key and putative genes involved with 2-methoxy-1,4-naphthoquinone (MNQ) biosynthesis in *Impatiens balsamina* L.**

Lian Chee Foong^1^, Jian Yi Chai^1^, Anthony Siong Hock Ho^1^, Brandon Pei Hui Yeo^2^, Yang Mooi Lim^3^ and Sheh May Tam^1^

**List of Supplementary Materials:**

**Supplemental Tables:**

Supplementary Table S1. Primer sequences of reference- and 20 selected target genes involved in four major secondary metabolite biosynthesis pathways used in qRT-PCR validation.

Supplementary Table S2. Retention time and corresponding peak areas of 2-hydroxy-1,4-naphthoquinone (lawsone) and 2-methoxy-1,4-naphthoquinone (MNQ) standards at five different concentrations (20-, 40-, 60-, 80- and 100 ppm) observed from RP-HPLC analyses.

Supplementary Table S3. Quantified amounts of 2-hydroxy-1,4-naphthoquinone (lawsone) and 2-methoxy-1,4-naphthoquinone (MNQ) extracted from different tissues of the pink multi-petal *Impatiens balsamina*.

Supplementary Table S4. Quality metrics of assembled transcripts and unigenes for the transcriptome of five different tissues of *Impatiens balsamina*.

Supplementary Table S5. Annotation summary of all *Impatiens balsamina* unigenes against seven public biological databases.

Supplementary Table S6. Total number of expressed unigenes present in each tissue of *Impatiens balsamina*.

Supplementary Table S7. Statistics of Gene Ontology (GO) and KEGG pathway enrichment for differential expressed genes (DEGs) from the *Impatiens balsamina* transcriptomes.

Supplementary Table S8. KEGG pathway enrichment for differential expressed genes (DEGs) of ten pairwise comparisons among five different tissues of *Impatiens balsamina*.

Supplementary Table S9. Gene expression profile of unigenes involved in the shikimate biosynthesis pathway among five different tissues of *Impatiens balsamina*.

Supplementary Table S10. Relative expression profile of genes from the 1,4-dihydroxy-2-naphthoate (DHNA) pathway in *Impatiens balsamina* between early- (E), mature- (M) and postbreaker (P) stages of capsule.

Supplementary Table S11. Nucleotide sequences of *Impatiens balsamina* candidate genes encoding three enzymes involved in the late steps of 2-methoxy-1,4-naphthoquinone (MNQ) biosynthesis.

Supplementary Table S12. Cycle threshold (Ct) values of reference genes for qRT-PCR analysis.

Supplementary Table S13. Fold change expressions of selected genes involved in selected secondary metabolite biosynthesis pathways obtained from qRT-PCR results compared with RNA-Seq data.

**Supplemental Figures:**

Supplementary Fig. S1A. Representative HPLC chromatographic profiles of standard lawsone (Std law) and extracts of different tissues of *Impatiens balsamina* with- and without spiking of 100 ppm lawsone.

Supplementary Fig. S1B. Representative HPLC chromatographic profiles of standard 2-methoxy-1,4-naphthoquinone (Std MNQ) and extracts of different tissues of *Impatiens balsamina* with- and without spiking of 100 ppm MNQ.

Supplementary Fig. S2. Linear calibration curves of two standard compounds, (A) 2-hydroxy-1,4-naphthoquinone (lawsone) and (B) 2-methoxy-1,4-naphthoquinone (MNQ), obtained from HPLC analysis.

Supplementary Fig. S3. Species distribution of NCBI NR annotated unigenes of *Impatiens balsamina* transcriptomes.

Supplementary Fig. S4. Gene ontology classification of the merged *Impatiens balsamina* transcriptomes from five different tissues (leaf, flower, early-, mature- and postbreaker stage capsules).

Supplementary Fig. S5. KOG categorisation of the merged *Impatiens balsamina* transcriptomes for the five different tissues (leaf, flower, early-, mature- and postbreaker stages of capsules).

Supplementary Fig. S6. KEGG pathway annotation of the merged *Impatiens balsamina* transcriptomes for the five different tissues (leaf, flower, early-, mature- and postbreaker stages of capsules).

Supplementary Fig. S7. Hierarchical clustering of differential expressed gene (DEG) expression profiles of *Impatiens balsamina* among pairwise tissue comparisons.

Supplementary Fig. S8. Hierarchical clustering of candidate genes encoding three enzymes postulated to be involved in the later steps of 2-methoxy-1,4-naphthoquinone (MNQ) biosynthesis.

Supplementary Fig. S9. Linear regression analysis of gene expression data obtained from qRT-PCR and RNA-Seq.

**Supplementary Table S1. Primer sequences of reference- and 20 selected target genes involved in four major secondary metabolite biosynthesis pathways used in qRT-PCR validation.**

| Gene symbol | Gene name [Enzyme Commission number] | Forward primer sequence (5' to 3') | Reverse primer sequence (5' to 3') |
| --- | --- | --- | --- |
| Reference genes/internal control | | | |
| *FBA* | Fructose-bisphosphate aldolase | CGTTATGCTGCCATTTCTCA | AGGGGATGCTTTTTCCTTGT |
| *EF1-α* | Elongation factor 1-alpha | ATCCGGTAGCATACGTGAGG | CGCTGAGATGTCCTCCTCTT |
| *UBC* | Ubiquitin-conjugated enzyme E2 | CGAGAAGCCTCCAACTGTTC | ACACCATTTCTTTCCGCAAC |
| Mevalonate (MVA) pathway | | | |
| *HMGS* | Hydroxymethylglutaryl-CoA synthase [EC:2.3.3.10] | CACCCCGTTTTCTTCTCTTG | TTCCCAACCAGGGAACTATG |
| *HMGR* | Hydroxymethylglutaryl-CoA reductase [EC:1.1.1.34] | GGGATCAAACGCAAGAATGT | GGAATTAGCAGCCAGTTTGG |
| *MVK* | Mevalonate kinase [EC:2.7.1.36] | ATGCTCAAACGAGACAAGCA | GCCGAGAATGCAACACATAA |
| *MVD* | Diphosphomevalonate decarboxylase [EC:4.1.1.33] | TTCCCTACTGCTGCTGGTTT | GTCGTCGTTCTTCCCCATAA |
| *IDI* | Isopentenyl-diphosphate Delta-isomerase [EC:5.3.3.2] | GAAGCTTTTGGACGAACTGG | TTCACGTCAGCAACCTCATC |
| 2-C-methyl-D-erythritol 4-phosphate (MEP) pathway | | | |
| *DXS* | 1-deoxy-D-xylulose-5-phosphate synthase [EC:2.2.1.7] | TCATCAGGCGTACCCACATA | TTGATCGTCCCTTCAAATCC |
| *DXR* | 1-deoxy-D-xylulose-5-phosphate reductoisomerase [EC:1.1.1.267] | GAACAGGGTGTGGTTGAGGT | GACCACCCGCAATTAGTGTT |
| *ISPD* | 2-C-methyl-D-erythritol 4-phosphate cytidylyltransferase [EC:2.7.7.60] | TGGACTTCAGGAGGCTGATT | CACCTGTGGTGTCTGCATTT |
| *ISPE* | 4-diphosphocytidyl-2-C-methyl-D-erythritol kinase [EC:2.7.1.148] | AGCTTTTCGGGTTCTTCCTT | GCCTCCGACAAGAAAACATC |
| *ISPF* | 2-C-methyl-D-erythritol 2,4-cyclodiphosphate synthase [EC:4.6.1.12] | AGCTTTTCGGGTTCTTCCTT | AAGCAATACATCCCCGTCTG |
| *ISPG* | (E)-4-hydroxy-3-methylbut-2-enyl-diphosphate synthase [EC:1.17.7.1 1.17.7.3] | CTGGCTAATCTCGGCAAAAG | ACGGTGTAGCACGCCTCTAT |

**Supplementary Table S1. continued**

| Gene symbol | Gene name [Enzyme Commission number] | Forward primer sequence (5' to 3') | Reverse primer sequence (5' to 3') |
| --- | --- | --- | --- |
| 2-C-methyl-D-erythritol 4-phosphate (MEP) pathway | | | |
| *ISPH* | 4-hydroxy-3-methylbut-2-en-1-yl diphosphate reductase [EC:1.17.7.4] | GCTTGCAGAGGCTTATGGTT | CAAACTGCTTCTTCCCATCC |
| Shikimate pathway | | | |
| *DAHPS* | 3-deoxy-7-phosphoheptulonate synthase [EC:2.5.1.54] | TCGTGACATGGGTCAGTGAT | CAGTGGGTGTGATAGCGAGA |
| *DHQS* | 3-dehydroquinate synthase [EC:4.2.3.4] | GATTGGAGAGAAGGCGAAGA | TGTTGTCCGCCTGTAAGTTG |
| *SK* | Shikimate kinase [EC:2.7.1.71] | TTACCGGCAACTCTCCATCT | GCCATCCGTGAACTCGTAAT |
| *EPSPS* | 3-phosphoshikimate 1-carboxyvinyltransferase [EC:2.5.1.19] | GTTGGGAAGGAATCATCCAA | CGCCAATTGGTCTCTCTCTC |
| *CS* | Chorismate synthase [EC:4.2.3.5] | GTTGTCCACCAAGGATTGCT | TGGAGCACCAGTTGTCAAAC |
| 1,4-dihydroxy-2-naphthoate (DHNA) pathway | | | |
| *PHYLLO* | Isochorismate synthase / 2-succinyl-5-enolpyruvyl-6-hydroxy-3-cyclohexene-1-carboxylate synthase / 2-succinyl-6-hydroxy-2,4-cyclohexadiene-1-carboxylate synthase / O-succinylbenzoate synthase [EC:5.4.4.2/2.2.1.9/4.2.99.20/4.2.1.113] | AGCTGATCGTCCACCTGAGT | TTAACATGAACGGGTCCACA |
| *AAE14* | Acyl-activating enzyme 14 [EC:6.2.1.26] | GGGGTGGAGACAGTAACGAA | TTTCCGGGAGGTGATAACAG |
| *MENB* | Naphthoate synthase [EC:4.1.3.36] | CTTTCCAGCACTGCCTTCTT | GTGAGCTTGGCTATGCCTTC |

**Supplementary Table S2. Retention time and corresponding peak areas of** **2-hydroxy-1,4-naphthoquinone (lawsone) and 2-methoxy-1,4-naphthoquinone (MNQ) standards at five different concentrations (20-, 40-, 60-, 80- and 100 ppm) observed from the RP-HPLC analyses.**

| Concentration (ppm) | Lawsone | | MNQ | |
| --- | --- | --- | --- | --- |
|  | Retention time (min) | Peak area (mAU) | Retention time (min) | Peak area (mAU) |
| 20 | 1.840 | 1814763 | 7.121 | 1466353 |
|  | 2.050 | 361440 |  |  |
|  | 2.291 | 263321 |  |  |
| 40 | 1.840 | 3649057 | 7.121 | 2724272 |
|  | 2.050 | 860441 |  |  |
|  | 2.291 | 603207 |  |  |
| 60 | 1.840 | 4987554 | 7.121 | 3998109 |
|  | 2.050 | 1585398 |  |  |
|  | 2.291 | 1078486 |  |  |
| 80 | 1.840 | 5959261 | 7.121 | 5413220 |
|  | 2.050 | 3131982 |  |  |
|  | 2.291 | 2046420 |  |  |
| 100 | 1.840 | NA | 7.121 | 6792732 |
|  | 2.050 | 5397237 |  |  |
|  | 2.291 | NA |  |  |

NA, not available

**Supplementary Table S3. Quantified amounts of 2-hydroxy-1,4-naphthoquinone (lawsone) and 2-methoxy-1,4-naphthoquinone (MNQ) extracted from different tissues of the pink multi-petal *Impatiens balsamina*.**

| Sample | Content of MNQ | | Content of lawsone | |
| --- | --- | --- | --- | --- |
|  | Average content mg g^-1^ extract ± SD | Average content mg g^-1^ dry weight ± SD | Average content mg g^-1^ extract ± SD | Average content mg g^-1^ dry weight ± SD |
|  |  |  |  |  |
| Mature leaf | ND | ND | 31.205 ± 7.894^a^ | 7.431 ± 1.915^a^ |
| Young leaf | ND | ND | 3.010 ± 0.229^b^ | 0.650 ± 0.039^b^ |
| Flower | ND | ND | 0.832 ± 0.305^c^ | 0.191 ± 0.067^c^ |
| Stem | ND | ND | 0.226 ± 0.086^de^ | 0.033 ±0.013^d^ |
| Root | ND | ND | 1.477 ± 0.274^f^ | 0.195 ±0.034^c^ |
| Early stage pericarp | 4.030 ± 1.482^a^ | 1.864 ± 0.697^a^ | 0.112 ± 0.082^d^ | 0.051 ± 0.035^d^ |
| Mature stage pericarp | 6.222 ± 0.641^b^ | 1.508 ± 0.189^a^ | 0.243 ±0.065^e^ | 0.060 ± 0.017^d^ |
| Third stage pericarp | 4.001 ± 1.084^a^ | 0.887 ± 0.244^b^ | 0.232 ± 0.090^de^ | 0.051 ± 0.020^d^ |
| Seed | ND | ND | ND | ND |
| Total naphthoquinones | 14.253 | 4.259 | 37.337 | 8.662 |

Average total contents (mg g^-1^) were calculated from multiple samples (with three to six biological replicates) analyzed from two HPLC analyses. *ND, not detected. Content of lawsone is the sum of the three lawsone tautomeric HPLC peaks (at the retention time of 1.844, 2.051, 2.284 min)
^a – f^ Labels with different letters indicate a significant difference (Games Howell test, p < 0.05) in the average contents between samples listed in the same column

**Supplementary Table S4. Quality metrics of assembled transcripts and unigenes for the transcriptome of five different tissues of *Impatiens balsamina*.**

| Sample | Transcripts | | | | Unigenes | | | | |
| --- | --- | --- | --- | --- | --- | --- | --- | --- | --- |
|  | Total Number | Total Length | Mean Length | Clean reads Q20 (%) ^1^ | Total number | Total length | Mean length | N50 | GC (%) |
| L1 | 63,550 | 57,850,362 | 910 | 99.13 | 50,886 | 51,250,735 | 1,007 | 1,581 | 42.88 |
| L2 | 64,474 | 58,262,651 | 903 | 99.09 | 50,436 | 51,189,240 | 1,014 | 1,601 | 42.90 |
| F1 | 48,405 | 45,854,139 | 947 | 99.09 | 38,574 | 40,708,321 | 1,055 | 1,601 | 43.34 |
| F2 | 48,298 | 46,026,874 | 952 | 99.07 | 38,305 | 40,661,607 | 1,061 | 1,602 | 43.40 |
| E1 | 60,839 | 57,530,934 | 945 | 99.10 | 48,221 | 51,058,115 | 1,058 | 1,647 | 43.23 |
| E2 | 62,316 | 58,271,522 | 935 | 98.88 | 47,178 | 50,302,591 | 1,066 | 1,661 | 43.24 |
| M1 | 56,200 | 52,189,782 | 928 | 99.03 | 45,377 | 47,092,248 | 1,037 | 1,585 | 42.87 |
| M2 | 57,714 | 53,381,240 | 924 | 98.88 | 44,135 | 45,975,397 | 1,041 | 1,588 | 42.94 |
| P1 | 66,082 | 59,884,059 | 906 | 99.25 | 52,114 | 54,160,304 | 1,039 | 1,671 | 42.50 |
| P2 | 67,950 | 62,282,956 | 916 | 99.22 | 50,730 | 52,134,608 | 1,027 | 1,647 | 42.51 |
| All-Unigene | - | - | - | - | 94,659 | 115,749,402 | 1,222 | 1,925 | 42.49 |

^1^Q20 (%) is the proportion of nucleotides in clean reads with quality value larger than 20. All-unigene refers to the clustered transcripts with consensus sequences in the merged transcriptome of the leaf (L), flower (F), early- (E), mature- (M) and postbreaker-(P) stage of capsules, after performing TGICL (Pertea et al., 2003).

**Supplementary Table S5. Annotation summary of all *Impatiens balsamina* unigenes against seven public biological databases.**

| Databases | Number of unigenes annotated | Percentage (%) |
| --- | --- | --- |
| NCBI Nr | 65,104 | 68.78 |
| NCBI Nt | 36,762 | 38.84 |
| Swissprot | 46,023 | 48.62 |
| KEGG | 51,098 | 53.98 |
| KOG | 55,114 | 58.22 |
| Interpro | 60,539 | 63.95 |
| GO | 24,445 | 25.82 |
| Intersection | 12,943 | 13.67 |
| Total annotated unigenes | 70,008 | 73.96 |
| Total unigenes | 94,659 | 100.00 |

Intersection refers to the number of unigenes which annotated by all the seven functional databases.

**Supplementary Table S6. Total number of expressed unigenes present in each tissue of *Impatiens balsamina.***

|  | Biological sample 1 | Biological sample 2 | Percentage (%, out of 94,659 unigenes) |
| --- | --- | --- | --- |
| Leaf | 59,368 | 59,648 | 62.87 |
| Flower | 50,084 | 49,741 | 52.73 |
| Early stage capsule | 59,102 | 59,637 | 62.72 |
| Mature stage capsule | 54,427 | 55,258 | 57.94 |
| Postbreaker stage capsule | 60,703 | 61,673 | 64.64 |

**Supplementary Table S7. Statistics of Gene Ontology (GO) and KEGG pathway enrichment for differential expressed genes (DEGs) from the *Impatiens balsamina* transcriptomes.**

| DEG Group | Total DEGs^a^ | Total DEGs with GO annotation | | | | Total DEGs with KEGG annotation | | | |
| --- | --- | --- | --- | --- | --- | --- | --- | --- | --- |
|  |  | Total number | % of total DEGs that with GO terms/total DEG | Up-regulated DEGs^a^ | Down-regulated DEGs^a^ | Total number | % of total DEGs that with KEGG terms/total DEG | Up-regulated DEGs^a^ | Down-regulated DEGs^a^ |
| F vs. P | 23,769 | 8,608 | 36.22 | 4,209 | 4,399 | 20,903 | 30.76 | 5,669 | 4,753 |
| L vs. P | 23,916 | 8,121 | 33.96 | 3,796 | 4,325 | 18,467 | 36.57 | 6,208 | 6,180 |
| L vs. F | 20,903 | 7,922 | 37.90 | 4,387 | 3,535 | 16,757 | 30.43 | 5,136 | 5,174 |
| E vs. P | 22,963 | 7,737 | 33.69 | 3,108 | 4,629 | 23,916 | 30.80 | 5,266 | 5,169 |
| F vs. M | 19,185 | 7,340 | 38.26 | 3,635 | 3,705 | 19,998 | 30.49 | 5,086 | 5,245 |
| F vs. E | 19,998 | 7,281 | 36.41 | 3,674 | 3,607 | 19,185 | 30.77 | 5,241 | 5,182 |
| L vs. M | 16,757 | 6,116 | 36.50 | 3,066 | 3,050 | 23,769 | 30.80 | 5,266 | 5,169 |
| L vs. E | 18,467 | 5,839 | 31.62 | 3,069 | 2,770 | 22,963 | 30.47 | 4,385 | 5,939 |
| E vs. M | 14,349 | 4,976 | 34.68 | 2,375 | 2,601 | 14,349 | 29.83 | 5,084 | 5,023 |
| M vs. P | 12,877 | 4,666 | 36.24 | 1,931 | 2,735 | 12,877 | 27.09 | 3,974 | 5,205 |
| Merged DEGs for all comparisons | 50,789 | 17,151 | 33.77 | - | - | 33,879 | 66.71 |  |  |

^a^DEGs were defined as the unigenes with significant log_2_ fold change ratio ≤ 2.0 in each comparison. L = leaf, F = flower, E = early-stage capsule, M = mature-stage capsule, P = postbreaker-stage capsule.

**Supplementary Table S8. KEGG pathway enrichment for differential expressed genes (DEGs) of ten pairwise comparisons among five different tissues of *Impatiens balsamina*.** **In the individual excel format “Supplementary Table S8”.**

**Supplementary Table S9. Gene expression profile of unigenes involved in the shikimate biosynthesis pathway among five different tissues of *Impatiens balsamina*. In the individual excel format “Supplementary Table S9”.**

**Supplementary Table S10. Relative expression profile of genes from the 1,4-dihydroxy-2-naphthoate (DHNA) pathway in *Impatiens balsamina* between early- (E), mature- (M) and postbreaker (P) stages of capsule.**

| Unigene ID | FPKM value | | | | | | Log_2_ fold change value | | |
| --- | --- | --- | --- | --- | --- | --- | --- | --- | --- |
|  | E1 | E2 | M1 | M2 | P1 | P2 | M vs E | P vs E | P vs M |
| CL4612.Contig2_All | 243.60 | 240.85 | 38.49 | 39.36 | 2.68 | 2.73 | -2.044* | -6.315* | -4.191* |
|  |  |  |  |  |  |  |  |  |  |
| CL4092.Contig3_All | 61.69 | 59.14 | 8.96 | 9.48 | 4.62 | 5.36 | -2.120* | -3.435* | -1.239* |
| CL4092.Contig2_All | 58.19 | 60.03 | 9.85 | 9.91 | 5.79 | 5.93 | -1.988* | -3.171* | -1.107* |
| Unigene24037_All | 20.45 | 22.19 | 4.50 | 4.82 | 1.36 | 2.04 | -1.595* | -3.469* | -1.778* |
| CL4092.Contig1_All | 8.97 | 7.43 | 0.81 | 0.54 | 0.00 | 0.18 | -3.006* | -3.647* | -2.413 |
| Unigene23373_All | 5.07 | 5.02 | 2.60 | 3.06 | 5.29 | 5.09 | -0.239 | 0.200 | 0.512 |
| Unigene23381_All | 0.48 | 0.58 | 0.00 | 0.34 | 0.00 | 0.47 | -0.774 | -0.770 | 0.052 |
| Unigene21821_All | 0.48 | 0.35 | 0.52 | 0.09 | 0.52 | 0.32 | 0.131 | 0.197 | 0.099 |
|  |  |  |  |  |  |  |  |  |  |
| CL1366.Contig2_All | 83.23 | 79.50 | 12.93 | 14.56 | 0.00 | 0.00 | -1.971* | -10.732* | -8.349* |
| CL1366.Contig1_All | 56.76 | 52.26 | 11.24 | 10.24 | 0.03 | 0.03 | -1.749* | -9.747* | -7.735* |
| CL1366.Contig3_All | 29.34 | 27.29 | 5.91 | 1.89 | 0.00 | 0.00 | -2.182* | -8.882* | -5.718* |
|  |  |  |  |  |  |  |  |  |  |
| CL643.Contig2_All | 934.22 | 890.81 | 118.33 | 109.26 | 1.37 | 0.68 | -2.408* | -9.620* | -7.119* |
| CL643.Contig1_All | 603.84 | 573.27 | 81.00 | 79.35 | 1.29 | 2.13 | -2.282* | -8.251* | -5.886* |
| Unigene8203_All | 584.63 | 552.21 | 84.00 | 79.73 | 2.88 | 3.48 | -2.200* | -7.295* | -5.010* |
| Unigene17346_All | 410.67 | 394.46 | 49.42 | 49.78 | 2.83 | 2.89 | -2.427* | -6.975* | -4.468* |
| Unigene17345_All | 319.73 | 309.51 | 40.51 | 40.14 | 1.75 | 2.53 | -2.370* | -7.045* | -4.592* |
| Unigene17344_All | 197.94 | 184.04 | 22.55 | 22.96 | 2.40 | 2.95 | -2.475* | -5.996* | -3.438* |
| Unigene17347_All | 164.46 | 157.07 | 23.39 | 24.83 | 0.11 | 0.52 | -2.139* | -8.534* | -6.223* |
| Unigene5523_All | 15.64 | 16.31 | 6.92 | 8.03 | 10 | 11.45 | -0.501 | -0.414 | 0.162 |
|  |  |  |  |  |  |  |  |  |  |
| CL6893.Contig7_All | 16.03 | 18.97 | 5.86 | 5.22 | 10.08 | 8.06 | -1.064* | -0.777 | 0.359 |
| CL6893.Contig3_All | 16.48 | 9.67 | 2.81 | 2.57 | 1.77 | 2.62 | -1.658* | -2.376* | -0.635 |
| CL6893.Contig5_All | 6.44 | 4.89 | 2.81 | 4.86 | 3.81 | 5.00 | 0.029 | -0.204 | -0.157 |
| CL6893.Contig6_All | 1.33 | 5.13 | 3.46 | 4.48 | 4.71 | 5.22 | 0.846 | 0.748 | -0.035 |
| CL6893.Contig8_All | 0.00 | 3.62 | 3.24 | 3.25 | 6.01 | 5.76 | 0.685 | 1.421 | 0.502 |
| CL6893.Contig2_All | 2.68 | 1.08 | 1.03 | 0.66 | 0.02 | 0.63 | -0.503 | -1.859 | -1.200 |
| CL6893.Contig4_All | 4.09 | 0.00 | 0.28 | 0.00 | 0.00 | 0.00 | -0.702 | -3.241 | -1.332 |
| CL6893.Contig1_All | 0.83 | 0.55 | 1.90 | 0.16 | 1.29 | 1.96 | 0.923 | 1.314 | 0.229 |

Log_2_ fold-change values were obtained based on normalised DESeq2 counts in the respective DEG pairwise comparisons. Asterisk symbol (*) indicates significant up- or down-regulation based on Fold Change ≥ 2.00 and Adjusted P-value ≤ 0.05

**Supplementary Table S11. Nucleotide sequences of *Impatiens balsamina* candidate genes encoding three enzymes involved in the late steps of 2-methoxy-1,4-naphthoquinone (MNQ) biosynthesis. In the individual excel format “Supplementary Table S11”.**

**Supplementary Table S12.** **Cycle threshold (Ct) values of reference genes for qRT-PCR analysis.**

| Tissue part of *Impatiens balsamina* | Ct mean value of reference gene* | | | Average Ct value |
| --- | --- | --- | --- | --- |
|  | *FBA* | *EF1-α* | *UBC* |  |
| Leaf | 20.19 | 21.78 | 18.96 | 20.31 |
| Flower | 20.64 | 21.28 | 18.02 | 19.98 |
| Early stage capsule | 19.24 | 20.55 | 18.75 | 19.51 |
| Mature stage capsule | 21.64 | 21.17 | 22.89 | 21.90 |
| Postbreaker stage capsule | 19.11 | 18.66 | 18.48 | 18.75 |

*Each Ct mean value obtained from two biological replicates and included in each batch of qRT-PCR run. Refer Table S1 for abbreviation of reference gene names.

**Supplementary Table S13. Fold change expressions of selected genes involved in selected secondary metabolite biosynthesis pathways obtained from qRT-PCR results compared with RNA-Seq data.**

| Gene symbol | Tissue sample | Cq (ref. genes) | Cq (sample) | ∆Cq | ∆Cq expression | Mean ∆Cq expression | ∆Cq expression std. dev. | ∆∆Cq Expression | ∆∆Cq Expression std. dev. | Log2 ∆∆Cq Expression | NGS (Log_2_ fold change)^a^ | NGS (fold change)^a^ |
| --- | --- | --- | --- | --- | --- | --- | --- | --- | --- | --- | --- | --- |
| mevalonate (MVA) pathway | | | | | | | | | | | | |
| *HMGS*  (Unigene20310_All) | L | 20.31 | 22.24 | 1.93 | 0.262 | 0.186 | 0.096 | 1.000 | 0.515 | 0.000 | 0.000 | 1.000 |
|  |  | 20.31 | 23.98 | 3.67 | 0.079 |  |  |  |  |  |  |  |
|  |  | 20.31 | 22.52 | 2.21 | 0.216 |  |  |  |  |  |  |  |
|  | E | 19.51 | 20.63 | 1.12 | 0.461 | 0.625 | 0.143 | 3.366 | 0.769 | 1.751 | 1.056 | 2.079 |
|  |  | 19.51 | 20.05 | 0.54 | 0.690 |  |  |  |  |  |  |  |
|  |  | 19.51 | 19.98 | 0.47 | 0.724 |  |  |  |  |  |  |  |
|  | M | 21.90 | 22.64 | 0.74 | 0.598 | 0.597 | 0.010 | 3.213 | 0.056 | 1.684 | 1.093 | 2.134 |
|  |  | 21.90 | 22.62 | 0.72 | 0.606 |  |  |  |  |  |  |  |
|  |  | 21.90 | 22.67 | 0.77 | 0.586 |  |  |  |  |  |  |  |
| *HMGR* (CL1289.Contig6_All) | E | 19.51 | 20.07 | 0.56 | 0.680 | 0.723 | 0.074 | 1.000 | 0.103 | 0.000 | 0.000 | 1.000 |
|  |  | 19.51 | 19.82 | 0.31 | 0.809 |  |  |  |  |  |  |  |
|  |  | 19.51 | 20.07 | 0.56 | 0.680 |  |  |  |  |  |  |  |
|  | M | 21.90 | 18.54 | -3.36 | 10.256 | 14.462 | 3.730 | 20.000 | 5.158 | 4.322 | 2.561 | 5.902 |
|  |  | 21.90 | 17.78 | -4.12 | 17.368 |  |  |  |  |  |  |  |
|  |  | 21.90 | 17.92 | -3.98 | 15.762 |  |  |  |  |  |  |  |
|  | P | 18.75 | 19.17 | 0.42 | 0.747 | 0.824 | 0.067 | 1.139 | 0.092 | 0.188 | 2.433 | 5.399 |
|  |  | 18.75 | 18.98 | 0.23 | 0.853 |  |  |  |  |  |  |  |
|  |  | 18.75 | 18.95 | 0.20 | 0.871 |  |  |  |  |  |  |  |
| *MVK* (Unigene27897_All) | L | 20.31 | 23.36 | 3.05 | 0.121 | 0.077 | 0.038 | 1.000 | 0.498 | 0.000 | 0.000 | 1.000 |
|  |  | 20.31 | 24.47 | 4.16 | 0.056 |  |  |  |  |  |  |  |
|  |  | 20.31 | 24.54 | 4.23 | 0.053 |  |  |  |  |  |  |  |

^a^ NGS refers to the relative gene expression profile (represented in log_2_ fold change and fold change) obtained from next generation sequencing of transcriptome in this study.

Supplementary Table S13. continued

| Gene symbol | Tissue sample | Cq (ref. genes) | Cq (sample) | ∆Cq | ∆Cq expression | Mean ∆Cq expression | ∆Cq expression std. dev. | ∆∆Cq Expression | ∆∆Cq Expression std. dev. | Log2 ∆∆Cq Expression | NGS (Log_2_ fold change)^a^ | NGS (fold change)^a^ |
| --- | --- | --- | --- | --- | --- | --- | --- | --- | --- | --- | --- | --- |
| *MVK* (Unigene27897_All) | E | 19.51 | 22.19 | 2.68 | 0.156 | 0.119 | 0.033 | 1.554 | 0.425 | 0.636 | 1.122 | 2.176 |
|  |  | 19.51 | 22.77 | 3.26 | 0.105 |  |  |  |  |  |  |  |
|  |  | 19.51 | 22.89 | 3.38 | 0.096 |  |  |  |  |  |  |  |
|  | P | 18.75 | 23.34 | 4.59 | 0.042 | 0.072 | 0.027 | 0.941 | 0.347 | -0.087 | -0.088 | 0.941 |
|  |  | 18.75 | 22.24 | 3.49 | 0.089 |  |  |  |  |  |  |  |
|  |  | 18.75 | 22.29 | 3.54 | 0.086 |  |  |  |  |  |  |  |
| *MVD* (CL8709.Contig1_All) | L | 20.13 | 24.31 | 4.18 | 0.055 | 0.051 | 0.009 | 1.000 | 0.184 | 0.000 | 0.000 | 1.000 |
|  |  | 20.13 | 24.76 | 4.63 | 0.040 |  |  |  |  |  |  |  |
|  |  | 20.13 | 24.24 | 4.11 | 0.058 |  |  |  |  |  |  |  |
|  | F | 19.98 | 24.05 | 4.07 | 0.060 | 0.060 | 0.009 | 1.174 | 0.167 | 0.232 | 0.325 | 1.252 |
|  |  | 19.98 | 24.25 | 4.27 | 0.052 |  |  |  |  |  |  |  |
|  |  | 19.98 | 23.84 | 3.86 | 0.069 |  |  |  |  |  |  |  |
|  | M | 21.90 | 24.5 | 2.60 | 0.165 | 0.152 | 0.016 | 2.968 | 0.314 | 1.570 | 1.539 | 2.906 |
|  |  | 21.90 | 24.57 | 2.67 | 0.157 |  |  |  |  |  |  |  |
|  |  | 21.90 | 24.8 | 2.90 | 0.134 |  |  |  |  |  |  |  |
| *IDI* (CL4464.Contig1_All) | L | 20.13 | 22.32 | 2.19 | 0.219 | 0.218 | 0.005 | 1.000 | 0.025 | 0.000 | 0.000 | 1.000 |
|  |  | 20.13 | 22.37 | 2.24 | 0.212 |  |  |  |  |  |  |  |
|  |  | 20.13 | 22.3 | 2.17 | 0.222 |  |  |  |  |  |  |  |
|  | M | 21.90 | 18.61 | -3.29 | 9.770 | 8.830 | 1.998 | 40.564 | 9.178 | 5.342 | 2.956 | 7.762 |
|  |  | 21.90 | 19.19 | -2.71 | 6.536 |  |  |  |  |  |  |  |
|  |  | 21.90 | 18.55 | -3.35 | 10.185 |  |  |  |  |  |  |  |
|  | P | 18.75 | 19.28 | 0.53 | 0.693 | 0.768 | 0.088 | 3.529 | 0.404 | 1.819 | 2.398 | 5.271 |
|  |  | 18.75 | 18.96 | 0.21 | 0.865 |  |  |  |  |  |  |  |
|  |  | 18.75 | 19.17 | 0.42 | 0.747 |  |  |  |  |  |  |  |

Supplementary Table S13. continued

| Gene symbol | Tissue sample | Cq (ref. genes) | Cq (sample) | ∆Cq | ∆Cq expression | Mean ∆Cq expression | ∆Cq expression std. dev. | ∆∆Cq Expression | ∆∆Cq Expression std. dev. | Log2 ∆∆Cq Expression | NGS (Log_2_ fold change)^a^ | NGS (fold change)^a^ |
| --- | --- | --- | --- | --- | --- | --- | --- | --- | --- | --- | --- | --- |
| 2-C-methyl-D-erythritol 4-phosphate (MEP) pathway | | | | | | | | | | | | |
| *DXS* (Unigene4794_All) | F | 19.98 | 26.16 | 6.18 | 0.014 | 0.011 | 0.002 | 1.000 | 0.219 | 0.000 | 0.000 | 1.000 |
|  |  | 19.98 | 26.67 | 6.69 | 0.010 |  |  |  |  |  |  |  |
|  |  | 19.98 | 26.69 | 6.71 | 0.010 |  |  |  |  |  |  |  |
|  | E | 19.51 | 22.08 | 2.57 | 0.169 | 0.164 | 0.016 | 14.900 | 1.470 | 3.897 | 2.195 | 4.580 |
|  |  | 19.51 | 22.01 | 2.50 | 0.177 |  |  |  |  |  |  |  |
|  |  | 19.51 | 22.29 | 2.78 | 0.146 |  |  |  |  |  |  |  |
|  | M | 21.90 | 25.56 | 3.66 | 0.079 | 0.079 | 0.005 | 7.171 | 0.471 | 2.842 | 3.157 | 8.920 |
|  |  | 21.90 | 25.47 | 3.57 | 0.084 |  |  |  |  |  |  |  |
|  |  | 21.90 | 25.66 | 3.76 | 0.074 |  |  |  |  |  |  |  |
| *DXR* (CL4760.Contig1_All) | F | 19.98 | 23.89 | 3.91 | 0.067 | 0.058 | 0.017 | 1.000 | 0.297 | 0.000 | 0.000 | 1.000 |
|  |  | 19.98 | 24.69 | 4.71 | 0.038 |  |  |  |  |  |  |  |
|  |  | 19.98 | 23.83 | 3.85 | 0.069 |  |  |  |  |  |  |  |
|  | E | 19.51 | 22.39 | 2.88 | 0.136 | 0.162 | 0.042 | 2.798 | 0.724 | 1.484 | 0.747 | 1.679 |
|  |  | 19.51 | 22.35 | 2.84 | 0.140 |  |  |  |  |  |  |  |
|  |  | 19.51 | 21.76 | 2.25 | 0.211 |  |  |  |  |  |  |  |
|  | M | 21.90 | 24.64 | 2.74 | 0.150 | 0.192 | 0.040 | 3.311 | 0.685 | 1.727 | 0.608 | 1.524 |
|  |  | 21.90 | 24.03 | 2.13 | 0.228 |  |  |  |  |  |  |  |
|  |  | 21.90 | 24.23 | 2.33 | 0.199 |  |  |  |  |  |  |  |
| *ispD* (Unigene8293_All) | L | 20.13 | 24.41 | 4.28 | 0.051 | 0.050 | 0.004 | 1.000 | 0.077 | 0.000 | 0.000 | 1.000 |
|  |  | 20.13 | 24.38 | 4.25 | 0.053 |  |  |  |  |  |  |  |
|  |  | 20.13 | 24.59 | 4.46 | 0.045 |  |  |  |  |  |  |  |
|  | M | 21.90 | 23.41 | 1.51 | 0.351 | 0.326 | 0.025 | 6.550 | 0.498 | 2.712 | 0.265 | 1.202 |
|  |  | 21.90 | 23.51 | 1.61 | 0.327 |  |  |  |  |  |  |  |
|  |  | 21.90 | 23.63 | 1.73 | 0.301 |  |  |  |  |  |  |  |

Supplementary Table S13. continued

| Gene symbol | Tissue sample | Cq (ref. genes) | Cq (sample) | ∆Cq | ∆Cq expression | Mean ∆Cq expression | ∆Cq expression std. dev. | ∆∆Cq Expression | ∆∆Cq Expression std. dev. | Log2 ∆∆Cq Expression | NGS (Log_2_ fold change)^a^ | NGS (fold change)^a^ |
| --- | --- | --- | --- | --- | --- | --- | --- | --- | --- | --- | --- | --- |
| *ispD* (Unigene8293_All) | P | 18.75 | 25.67 | 6.92 | 0.008 | 0.008 | 0.001 | 0.159 | 0.022 | -2.654 | -2.418 | 0.187 |
|  |  | 18.75 | 25.97 | 7.22 | 0.007 |  |  |  |  |  |  |  |
|  |  | 18.75 | 25.58 | 6.83 | 0.009 |  |  |  |  |  |  |  |
| *ispE* (CL1700.Contig2_All) | P | 18.75 | 22.75 | 4.00 | 0.063 | 0.059 | 0.004 | 1.000 | 0.076 | 0.000 | 0.000 | 1.000 |
|  |  | 18.75 | 22.96 | 4.21 | 0.054 |  |  |  |  |  |  |  |
|  |  | 18.75 | 22.79 | 4.04 | 0.061 |  |  |  |  |  |  |  |
|  | M | 21.90 | 23.46 | 1.56 | 0.339 | 0.318 | 0.021 | 5.380 | 0.354 | 2.428 | 2.627 | 6.179 |
|  |  | 21.90 | 23.65 | 1.75 | 0.297 |  |  |  |  |  |  |  |
|  |  | 21.90 | 23.55 | 1.65 | 0.318 |  |  |  |  |  |  |  |
|  | E | 19.51 | 22.15 | 2.64 | 0.161 | 0.196 | 0.032 | 3.313 | 0.545 | 1.728 | 1.069 | 2.097 |
|  |  | 19.51 | 21.82 | 2.31 | 0.202 |  |  |  |  |  |  |  |
|  |  | 19.51 | 21.67 | 2.16 | 0.224 |  |  |  |  |  |  |  |
| *ispF* (Unigene942_All) | L | 20.13 | 23.2 | 3.07 | 0.119 | 0.128 | 0.014 | 1.000 | 0.113 | 0.000 | 0.000 | 1.000 |
|  |  | 20.13 | 23.19 | 3.06 | 0.120 |  |  |  |  |  |  |  |
|  |  | 20.13 | 22.92 | 2.79 | 0.145 |  |  |  |  |  |  |  |
|  | M | 21.90 | 27.37 | 5.47 | 0.023 | 0.011 | 0.010 | 0.087 | 0.078 | -3.522 | -1.467 | 0.362 |
|  |  | 21.90 | 29.16 | 7.26 | 0.007 |  |  |  |  |  |  |  |
|  |  | 21.90 | 29.75 | 7.85 | 0.004 |  |  |  |  |  |  |  |
|  | P | 18.75 | 24.08 | 5.33 | 0.025 | 0.011 | 0.012 | 0.089 | 0.093 | -3.489 | -1.344 | 0.394 |
|  |  | 18.75 | 25.97 | 7.22 | 0.007 |  |  |  |  |  |  |  |
|  |  | 18.75 | 27.34 | 8.59 | 0.003 |  |  |  |  |  |  |  |
| *ispG* (CL10043.Contig2_All) | L | 20.13 | 21.5 | 1.37 | 0.387 | 0.353 | 0.052 | 1.000 | 0.147 | 0.000 | 0.000 | 1.000 |
|  |  | 20.13 | 21.53 | 1.40 | 0.379 |  |  |  |  |  |  |  |
|  |  | 20.13 | 21.9 | 1.77 | 0.293 |  |  |  |  |  |  |  |

Supplementary Table S13. continued

| Gene symbol | Tissue sample | Cq (ref. genes) | Cq (sample) | ∆Cq | ∆Cq expression | Mean ∆Cq expression | ∆Cq expression std. dev. | ∆∆Cq Expression | ∆∆Cq Expression std. dev. | Log2 ∆∆Cq Expression | NGS (Log_2_ fold change)^a^ | NGS (fold change)^a^ |
| --- | --- | --- | --- | --- | --- | --- | --- | --- | --- | --- | --- | --- |
| *ispG* (CL10043.Contig2_All) | E | 19.51 | 22.81 | 3.30 | 0.102 | 0.125 | 0.027 | 0.355 | 0.078 | -1.495 | -1.023 | 0.492 |
|  |  | 19.51 | 22.2 | 2.69 | 0.155 |  |  |  |  |  |  |  |
|  |  | 19.51 | 22.59 | 3.08 | 0.119 |  |  |  |  |  |  |  |
|  | P | 18.75 | 22.77 | 4.02 | 0.062 | 0.072 | 0.017 | 0.204 | 0.047 | -2.290 | -1.345 | 0.394 |
|  |  | 18.75 | 22.2 | 3.45 | 0.092 |  |  |  |  |  |  |  |
|  |  | 18.75 | 22.73 | 3.98 | 0.063 |  |  |  |  |  |  |  |
| *ispH* (Unigene24474_All) | L | 20.13 | 19.07 | -1.06 | 2.085 | 2.042 | 0.409 | 1.000 | 0.200 | 0.000 | 0.000 | 1.000 |
|  |  | 20.13 | 19.44 | -0.69 | 1.613 |  |  |  |  |  |  |  |
|  |  | 20.13 | 18.85 | -1.28 | 2.428 |  |  |  |  |  |  |  |
|  | E | 19.51 | 20.41 | 0.90 | 0.537 | 0.922 | 0.525 | 0.452 | 0.257 | -1.147 | -1.082 | 0.472 |
|  |  | 19.51 | 18.91 | -0.60 | 1.520 |  |  |  |  |  |  |  |
|  |  | 19.51 | 20.01 | 0.50 | 0.709 |  |  |  |  |  |  |  |
|  | M | 21.90 | 24.04 | 2.14 | 0.227 | 0.410 | 0.417 | 0.201 | 0.204 | -2.316 | -1.344 | 0.394 |
|  |  | 21.90 | 25.01 | 3.11 | 0.116 |  |  |  |  |  |  |  |
|  |  | 21.90 | 22.07 | 0.17 | 0.888 |  |  |  |  |  |  |  |

Supplementary Table S13. continued

| Gene symbol | Tissue sample | Cq (ref. genes) | Cq (sample) | ∆Cq | ∆Cq expression | Mean ∆Cq expression | ∆Cq expression std. dev. | ∆∆Cq Expression | ∆∆Cq Expression std. dev. | Log2 ∆∆Cq Expression | NGS (Log_2_ fold change)^a^ | | | NGS (fold change)^a^ |
| --- | --- | --- | --- | --- | --- | --- | --- | --- | --- | --- | --- | --- | --- | --- |
| Shikimate pathway | | | | | | | | | | | | | | |
| *DAHPS* (CL3764.Contig1_All) | F | 19.98 | 18.8 | -1.18 | 2.266 | 2.156 | 0.129 | 1.000 | 0.060 | 0.000 | | 0.000 | 1.000 | |
|  |  | 19.98 | 18.85 | -1.13 | 2.189 |  |  |  |  |  | |  |  | |
|  |  | 19.98 | 18.97 | -1.01 | 2.014 |  |  |  |  |  | |  |  | |
|  | L | 20.13 | 22.77 | 2.64 | 0.160 | 0.196 | 0.033 | 0.091 | 0.016 | -3.461 | | -6.124 | 0.014 | |
|  |  | 20.13 | 22.27 | 2.14 | 0.227 |  |  |  |  |  | |  |  | |
|  |  | 20.13 | 22.45 | 2.32 | 0.200 |  |  |  |  |  | |  |  | |
|  | E | 19.51 | 22 | 2.49 | 0.179 | 0.152 | 0.024 | 0.070 | 0.011 | -3.830 | | -5.389 | 0.024 | |
|  |  | 19.51 | 22.43 | 2.92 | 0.132 |  |  |  |  |  | |  |  | |
|  |  | 19.51 | 22.31 | 2.80 | 0.144 |  |  |  |  |  | |  |  | |
| *DHQS* (Unigene27756_All) | F | 19.98 | 23.41 | 3.43 | 0.093 | 0.082 | 0.011 | 1.000 | 0.129 | 0.000 | | 0.000 | 1.000 | |
|  |  | 19.98 | 23.61 | 3.63 | 0.081 |  |  |  |  |  | |  |  | |
|  |  | 19.98 | 23.78 | 3.80 | 0.072 |  |  |  |  |  | |  |  | |
|  | E | 19.51 | 24.52 | 5.01 | 0.031 | 0.036 | 0.013 | 0.440 | 0.158 | -1.186 | | -2.838 | 0.140 | |
|  |  | 19.51 | 24.77 | 5.26 | 0.026 |  |  |  |  |  | |  |  | |
|  |  | 19.51 | 23.82 | 4.31 | 0.051 |  |  |  |  |  | |  |  | |
|  | M | 21.90 | 23.27 | 1.37 | 0.386 | 0.317 | 0.066 | 3.876 | 0.808 | 1.955 | | 1.174 | 2.257 | |
|  |  | 21.90 | 23.59 | 1.69 | 0.310 |  |  |  |  |  | |  |  | |
|  |  | 21.90 | 23.87 | 1.97 | 0.255 |  |  |  |  |  | |  |  | |
| *SK* (CL5350.Contig1_All) | L | 20.13 | 24.52 | 4.39 | 0.048 | 0.043 | 0.004 | 1.000 | 0.100 | 0.000 | | 0.000 | 1.000 | |
|  |  | 20.13 | 24.66 | 4.53 | 0.043 |  |  |  |  |  | |  |  | |
|  |  | 20.13 | 24.81 | 4.68 | 0.039 |  |  |  |  |  | |  |  | |
|  | F | 19.98 | 26.02 | 6.04 | 0.015 | 0.014 | 0.002 | 0.333 | 0.057 | -1.587 | | -1.385 | 0.383 | |
|  |  | 19.98 | 25.91 | 5.93 | 0.016 |  |  |  |  |  | |  |  | |
|  |  | 19.98 | 26.4 | 6.42 | 0.012 |  |  |  |  |  | |  |  | |

Supplementary Table S13. continued

| Gene symbol | Tissue sample | Cq (ref. genes) | Cq (sample) | ∆Cq | ∆Cq expression | Mean ∆Cq expression | ∆Cq expression std. dev. | ∆∆Cq Expression | ∆∆Cq Expression std. dev. | Log2 ∆∆Cq Expression | NGS (Log_2_ fold change)^a^ | NGS (fold change)^a^ |
| --- | --- | --- | --- | --- | --- | --- | --- | --- | --- | --- | --- | --- |
| *SK* (CL5350.Contig1_All) | P | 18.75 | 23.35 | 4.60 | 0.041 | 0.033 | 0.007 | 0.752 | 0.173 | -0.411 | -0.046 | 0.969 |
|  |  | 18.75 | 23.89 | 5.14 | 0.028 |  |  |  |  |  |  |  |
|  |  | 18.75 | 23.9 | 5.15 | 0.028 |  |  |  |  |  |  |  |
| *EPSPS* (CL7586.Contig1_All) | F | 19.98 | 23.31 | 3.33 | 0.099 | 0.091 | 0.009 | 1.000 | 0.094 | 0.000 | 0.000 | 1.000 |
|  |  | 19.98 | 23.46 | 3.48 | 0.090 |  |  |  |  |  |  |  |
|  |  | 19.98 | 23.58 | 3.60 | 0.082 |  |  |  |  |  |  |  |
|  | E | 19.51 | 21.67 | 2.16 | 0.224 | 0.198 | 0.023 | 2.183 | 0.257 | 1.126 | 0.756 | 1.688 |
|  |  | 19.51 | 21.96 | 2.45 | 0.184 |  |  |  |  |  |  |  |
|  |  | 19.51 | 21.95 | 2.44 | 0.185 |  |  |  |  |  |  |  |
|  | M | 21.90 | 24.44 | 2.54 | 0.172 | 0.165 | 0.007 | 1.825 | 0.076 | 0.868 | 0.365 | 1.288 |
|  |  | 21.90 | 24.56 | 2.66 | 0.158 |  |  |  |  |  |  |  |
|  |  | 21.90 | 24.49 | 2.59 | 0.166 |  |  |  |  |  |  |  |
| *CS* (Unigene17766_All) | L | 20.13 | 23.06 | 2.93 | 0.131 | 0.154 | 0.025 | 1.000 | 0.160 | 0.000 | 0.000 | 1.000 |
|  |  | 20.13 | 22.85 | 2.72 | 0.152 |  |  |  |  |  |  |  |
|  |  | 20.13 | 22.6 | 2.47 | 0.180 |  |  |  |  |  |  |  |
|  | F | 19.98 | 22.39 | 2.41 | 0.188 | 0.186 | 0.016 | 1.201 | 0.104 | 0.265 | 1.398 | 2.636 |
|  |  | 19.98 | 22.55 | 2.57 | 0.168 |  |  |  |  |  |  |  |
|  |  | 19.98 | 22.3 | 2.32 | 0.200 |  |  |  |  |  |  |  |
|  | M | 21.90 | 25.25 | 3.35 | 0.098 | 0.115 | 0.015 | 0.743 | 0.100 | -0.428 | -0.109 | 0.927 |
|  |  | 21.90 | 24.86 | 2.96 | 0.128 |  |  |  |  |  |  |  |
|  |  | 21.90 | 24.98 | 3.08 | 0.118 |  |  |  |  |  |  |  |

Supplementary Table S13. continued

| Gene symbol | Tissue sample | Cq (ref. genes) | Cq (sample) | ∆Cq | ∆Cq expression | Mean ∆Cq expression | ∆Cq expression std. dev. | ∆∆Cq Expression | ∆∆Cq Expression std. dev. | Log2 ∆∆Cq Expression | NGS (Log_2_ fold change)^a^ | NGS (fold change)^a^ |
| --- | --- | --- | --- | --- | --- | --- | --- | --- | --- | --- | --- | --- |
| 1,4-dihydroxy-2-naphthoate (DHNA) pathway | | | | | | | | | | | | |
| *PHYLLO* (CL4092.Contig3_All) | E | 19.51 | 22.04 | 2.53 | 0.174 | 0.163 | 0.009 | 1.000 | 0.055 | 0.000 | 0.000 | 1.000 |
|  |  | 19.51 | 22.17 | 2.66 | 0.159 |  |  |  |  |  |  |  |
|  |  | 19.51 | 22.18 | 2.67 | 0.158 |  |  |  |  |  |  |  |
|  | F | 19.98 | 26.79 | 6.81 | 0.009 | 0.008 | 0.001 | 0.051 | 0.009 | -4.306 | -2.750 | 0.149 |
|  |  | 19.98 | 26.73 | 6.75 | 0.009 |  |  |  |  |  |  |  |
|  |  | 19.98 | 27.23 | 7.25 | 0.007 |  |  |  |  |  |  |  |
|  | L | 20.13 | 27.11 | 6.98 | 0.008 | 0.009 | 0.001 | 0.055 | 0.006 | -4.179 | -3.041 | 0.121 |
|  |  | 20.13 | 26.79 | 6.66 | 0.010 |  |  |  |  |  |  |  |
|  |  | 20.13 | 26.89 | 6.76 | 0.009 |  |  |  |  |  |  |  |
| *AAE14* (CL1366.Contig2_All) | F | 19.98 | 25.39 | 5.41 | 0.024 | 0.019 | 0.005 | 1.000 | 0.246 | 0.000 | 0.000 | 1.000 |
|  |  | 19.98 | 25.73 | 5.75 | 0.019 |  |  |  |  |  |  |  |
|  |  | 19.98 | 26.11 | 6.13 | 0.014 |  |  |  |  |  |  |  |
|  | E | 19.51 | 21.12 | 1.61 | 0.329 | 0.310 | 0.047 | 16.483 | 2.517 | 4.043 | 3.449 | 10.919 |
|  |  | 19.51 | 21.48 | 1.97 | 0.256 |  |  |  |  |  |  |  |
|  |  | 19.51 | 21.05 | 1.54 | 0.345 |  |  |  |  |  |  |  |

Supplementary Table S13. continued

| Gene symbol | Tissue sample | Cq (ref. genes) | Cq (sample) | ∆Cq | ∆Cq expression | Mean ∆Cq expression | ∆Cq expression std. dev. | ∆∆Cq Expression | ∆∆Cq Expression std. dev. | Log2 ∆∆Cq Expression | NGS (Log_2_ fold change)^a^ | NGS (fold change)^a^ |
| --- | --- | --- | --- | --- | --- | --- | --- | --- | --- | --- | --- | --- |
| *AAE14* (CL1366.Contig2_All) | P | 18.75 | 29.79 | 11.04 | 0.000 | 0.000 | 0.000 | 0.024 | 0.007 | -5.366 | -7.714 | 0.005 |
|  |  | 18.75 | 30.36 | 11.61 | 0.000 |  |  |  |  |  |  |  |
|  |  | 18.75 | 29.52 | 10.77 | 0.001 |  |  |  |  |  |  |  |
| *menB* (CL643.Contig2_All) | F | 19.98 | 25.86 | 5.88 | 0.017 | 0.019 | 0.003 | 1.000 | 0.167 | 0.000 | 0.000 | 1.000 |
|  |  | 19.98 | 25.9 | 5.92 | 0.017 |  |  |  |  |  |  |  |
|  |  | 19.98 | 25.48 | 5.50 | 0.022 |  |  |  |  |  |  |  |
|  | E | 19.51 | 19.26 | -0.25 | 1.193 | 1.144 | 0.164 | 61.744 | 8.825 | 5.948 | 5.478 | 44.565 |
|  |  | 19.51 | 19.16 | -0.35 | 1.278 |  |  |  |  |  |  |  |
|  |  | 19.51 | 19.57 | 0.06 | 0.962 |  |  |  |  |  |  |  |
|  | P | 18.75 | 26.01 | 7.26 | 0.007 | 0.005 | 0.001 | 0.279 | 0.071 | -1.841 | -4.017 | 0.062 |
|  |  | 18.75 | 26.36 | 7.61 | 0.005 |  |  |  |  |  |  |  |
|  |  | 18.75 | 26.76 | 8.01 | 0.004 |  |  |  |  |  |  |  |

| **A **  2.272 min  2.054 min  1.838 min | **B**  ****  1.847 min  2.060 min  2.282 min |
| --- | --- |
| **C**  ****  2.283 min  2.057 min  1.847min | **D**  ****  2.278 min  2.050 min  1.841 min |
| **E**  ****  2.267 min  2.054 min  1.835 min | **F  **  1.842 min  2.057 min  2.258 min |
| **G**  ****  2.233 min  1.844 min | **H**  ****  2.105 min |
| **I**  2.102 min  **** | **J**  **** |
| **K**  ****  2.104 min | **L**  ****  2.288 min  1.843 min  2.054 min |
| **M**  ****  2.102 min | **N**  ****  2.104 min |
| **O**  ****  2.102 min |  |

**Supplementary Figure S1A. Representative HPLC chromatographic profiles of standard lawsone (Std law) and extracts of different tissues of *Impatiens balsamina* with- and without spiking of 100 ppm lawsone.** (A) Std Law 100 ppm, (B) Std Law 80 ppm, (C) Std Law 60ppm, (D) Std Law 40ppm, (E) Std Law 20ppm, (F) Mature leaves extract, (G) Mature leaves extract with spiking of 100 ppm, (H) Young leaves extract, (I) Roots extract, (J) Seeds extract, (K) Early stage pericarps extract, (L) Mature stage pericarps extract, (M) Postbreaker stage pericarps extract, (N) Flowers extract and (O) Stems extract of *I. balsamina*. Standard Lawsone was detected at the wavelength of 266 nm. Arrows point to lawsone (peak and RT) with corresponding spectra shown as insert. Y-axis denotes peak area (mAU), and x-axis denotes retention time (minutes).

| **A**  ****  7.379 min | **B**  ****  7.352 min |
| --- | --- |
| **C**  ****  7.349 min | **D**  ****  7.329 min |
| **E**  ****  7.368 min | **F**  ****  7.177 min |
| **G**  ****  7.134 min | **H**  ****  7.013 min |
| **I **  7.063 min | J   |
| **K**  **** | L  |
| M   | N  **** |
| O  **** |  |

**Supplementary Figure S1B. Representative HPLC chromatographic profiles of standard 2-methoxy-1,4-naphthoquinone (Std MNQ) and extracts of different tissues of *Impatiens balsamina*with- and without spiking of 100 ppm MNQ.**

**(A)** Std MNQ 100ppm, **(B)**Std MNQ 80ppm, **(C)**Std MNQ 60ppm,**(D)**Std MNQ 40ppm, **(E)**Std MNQ 20ppm, **(F)**Mature pericarps extract, **(G)**Mature pericarps extract, **(H)** Early stage pericarps extract, **(I)** Postbreaker stage pericarps extract, **(J)**Flowers extract, **(K)** Seeds extract, **(L)** Roots extract, **(M)** Stems extract, **(N)**Young leaves extract and **(O)**Mature leaves extract of *I. balsamina* with spiking of 100 ppm. Std MNQ, at the wavelength of 266 nm. Arrows point to the MNQ peak with retention time (RT) and corresponding spectra shown in insert. Y-axis denotes peak area (mAU) and x-axis denotes retention time (minutes).


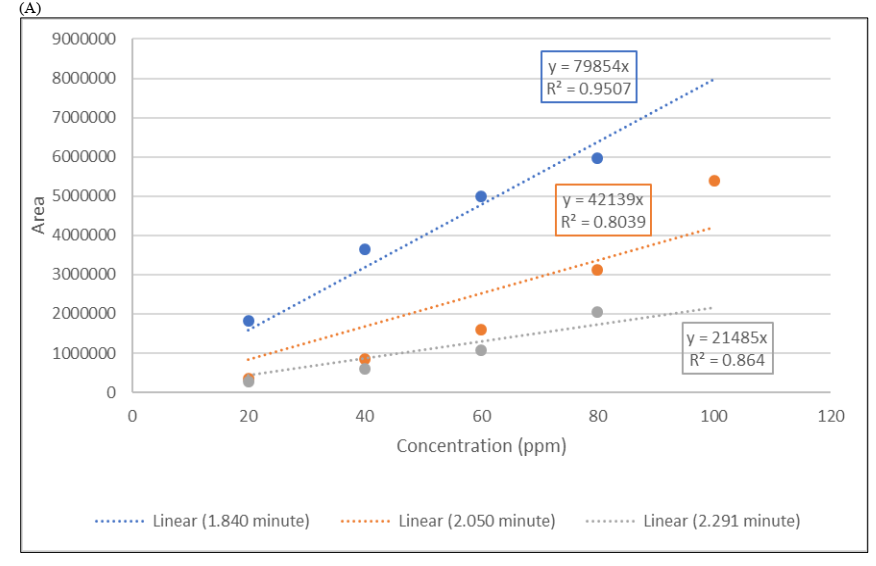


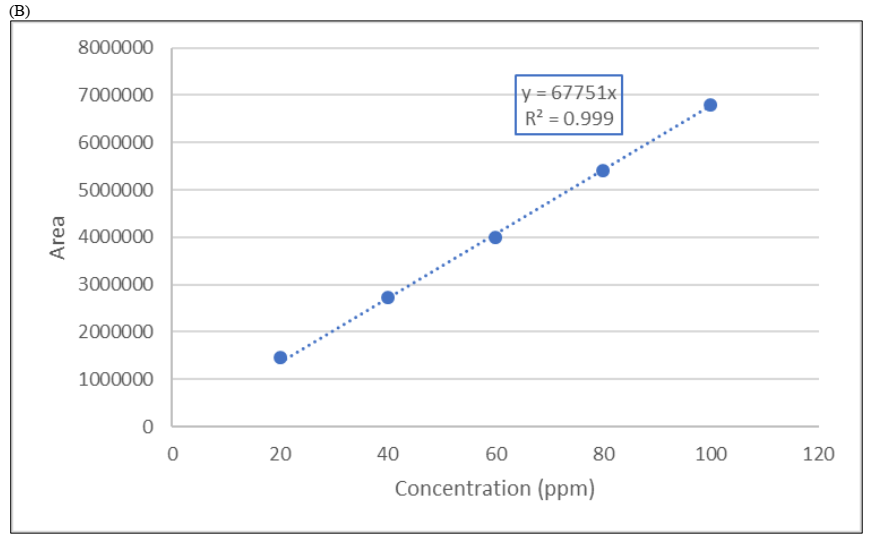


**Supplementary Figure S2. Linear calibration curves of two standard compounds, (A) 2-hydroxy-1,4-naphthoquinone (lawsone) and (B) 2-methoxy-1,4-naphthoquinone (MNQ), obtained from HPLC analysis.** Standard lawsone were detected at the retention times of 1.840 (blue), 2.050 (orange) and 2.291 (grey) minute (due to the three tautomeric forms of lawsone), while standard MNQ were detected at the retention time of 7.121 minute.


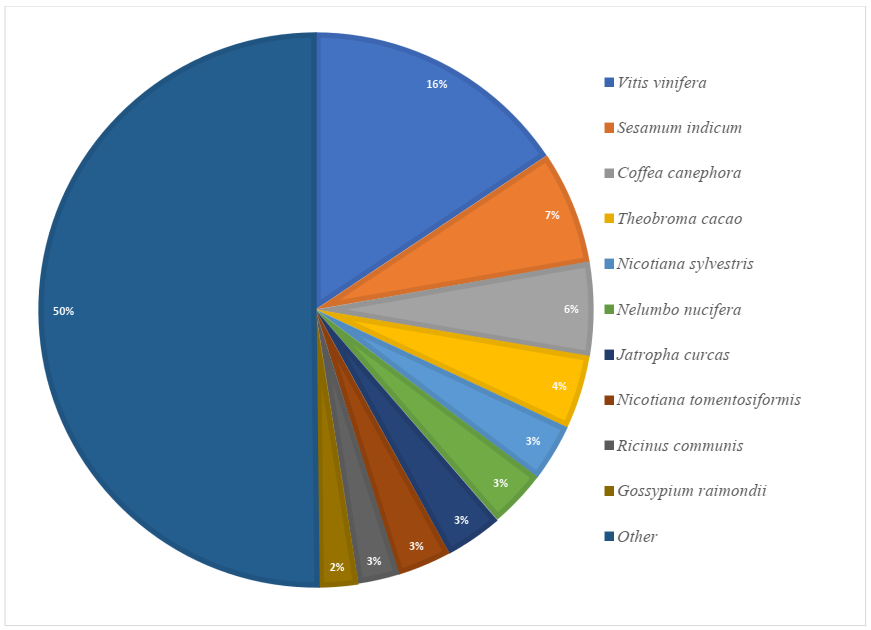


**Supplementary Figure S3. Species distribution of NCBI NR annotated unigenes of *Impatiens balsamina* transcriptomes.**


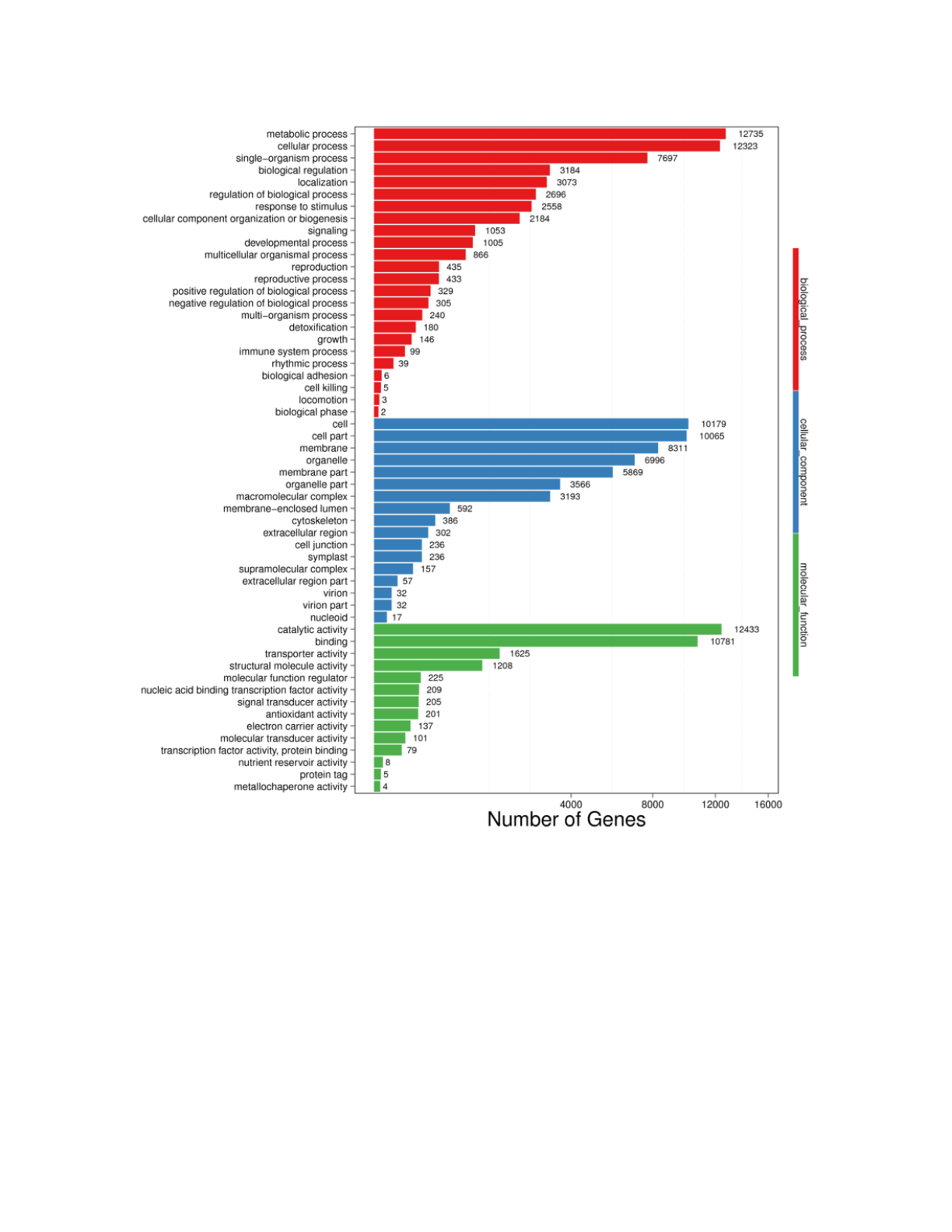


**Supplementary Figure S4. Gene ontology classification of the merged *Impatiens balsamina* transcriptomes from five different tissues (leaf, flower, early-, mature- and postbreaker stage capsules).**


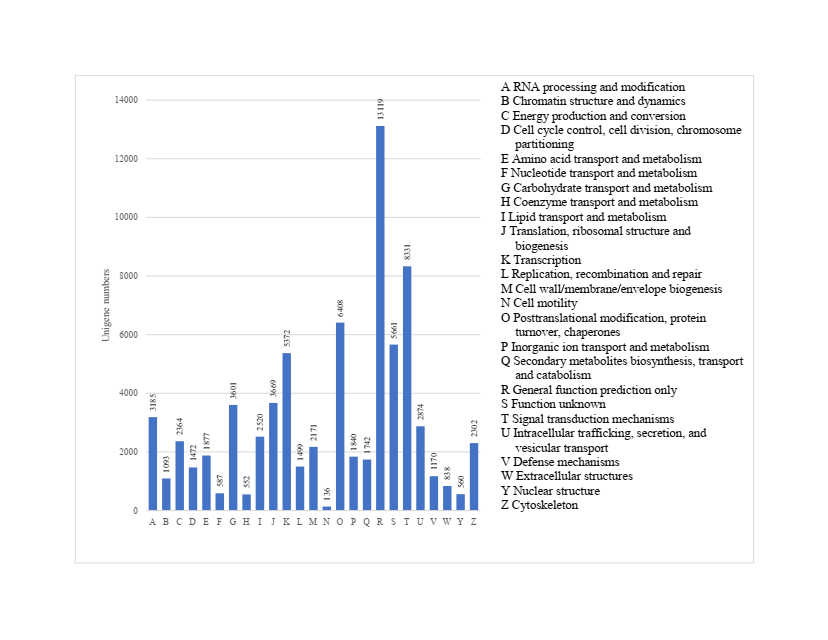


**Supplementary Figure S5. KOG categorisation of the merged *Impatiens balsamina* transcriptomes for the five different tissues (leaf, flower, early-, mature- and postbreaker stages of capsules).**


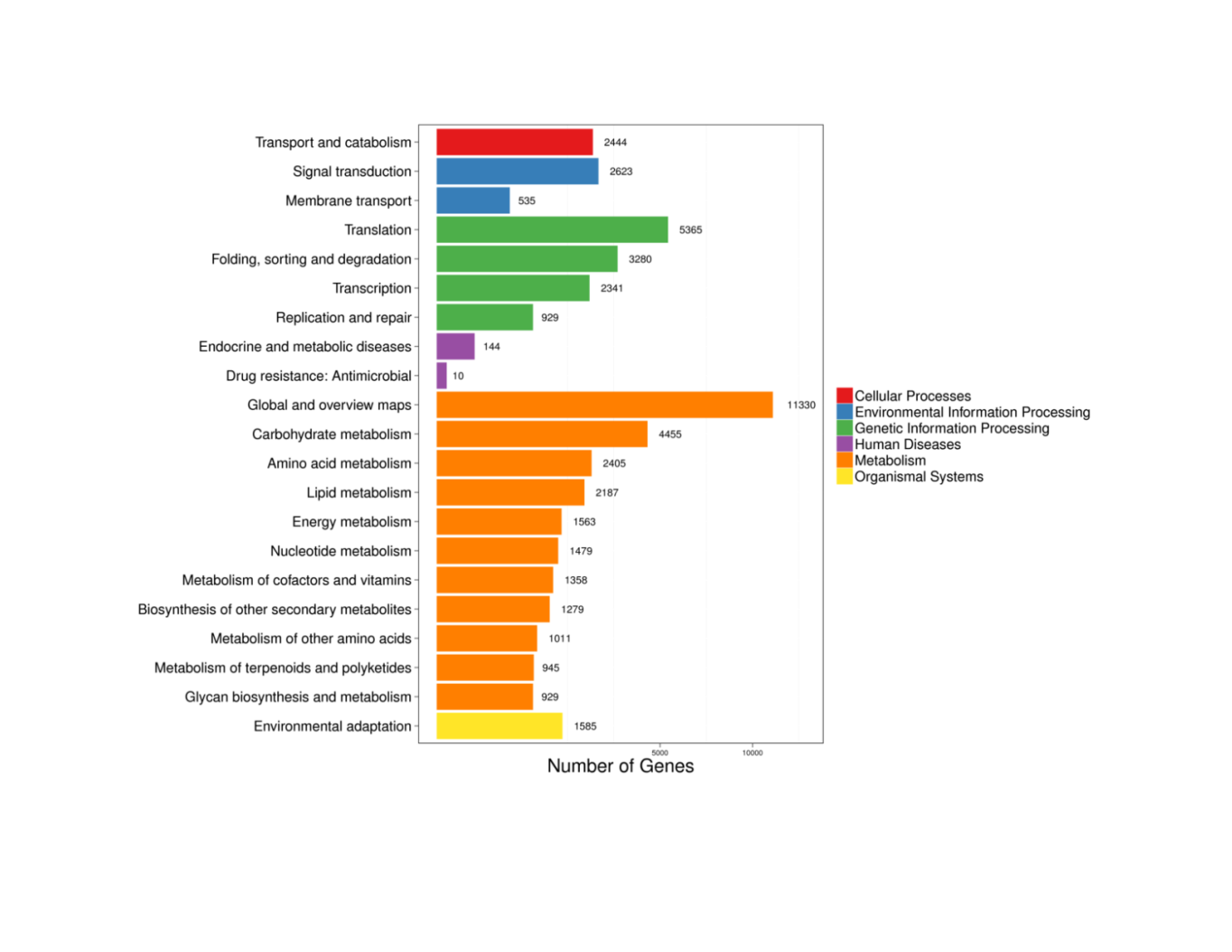


**Supplementary Figure S6. KEGG pathway annotation of the merged *Impatiens balsamina* transcriptomes for the five different tissues (leaf, flower, early-, mature- and postbreaker stages of capsules).**


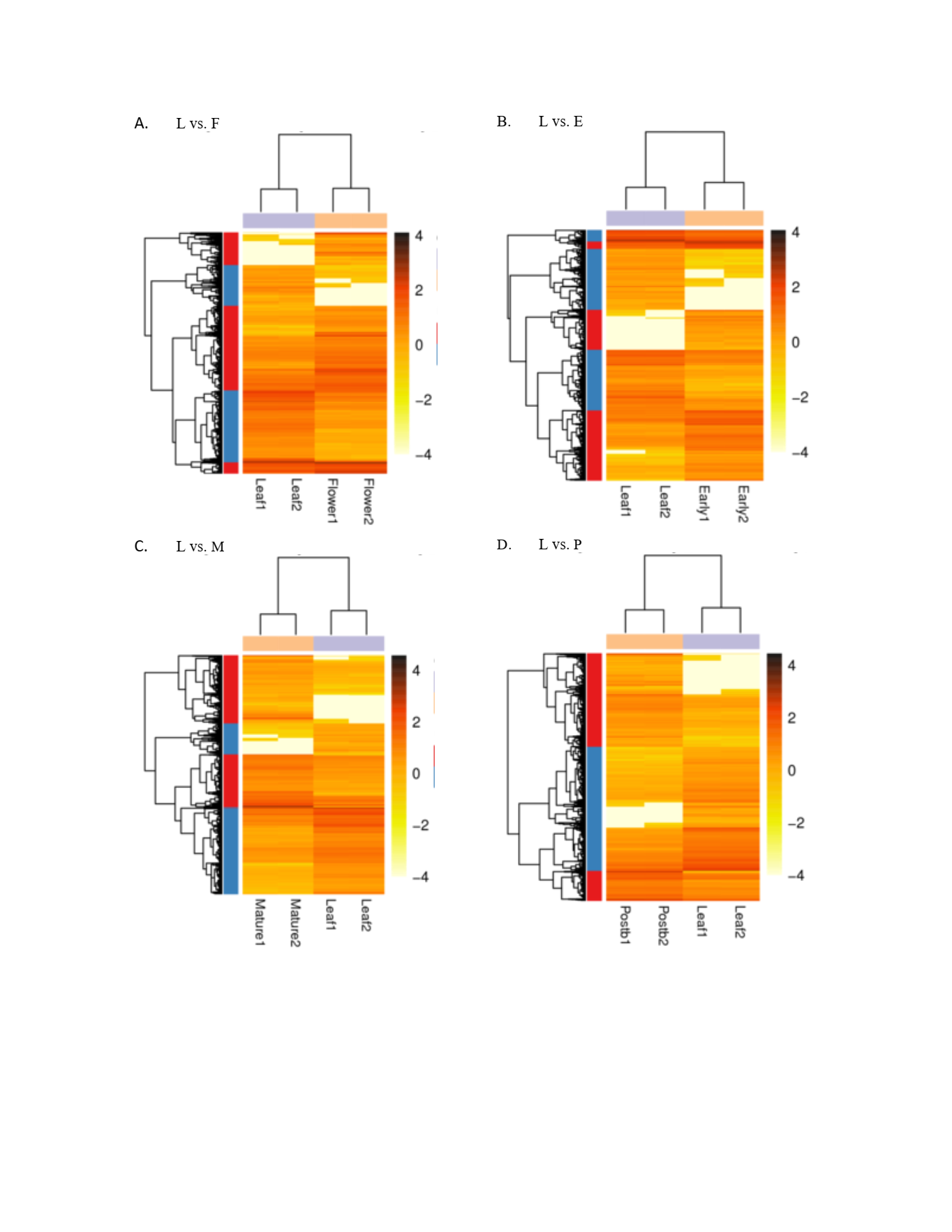

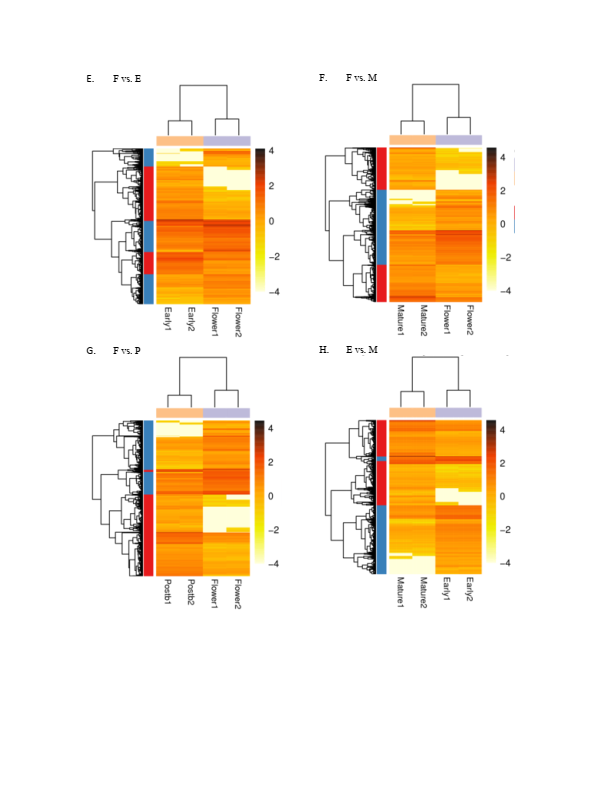

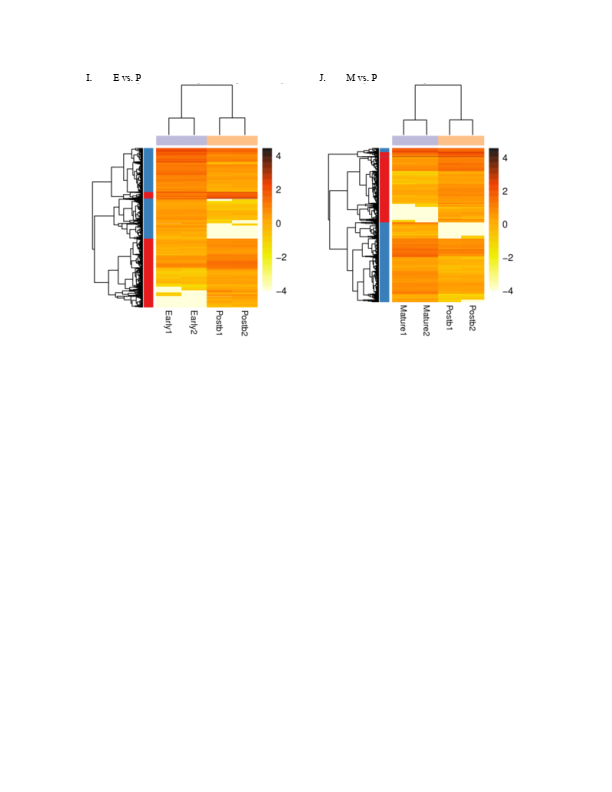


**Supplementary Figure S7. Hierarchical clustering of differential expressed gene (DEG) expression profiles of *Impatiens balsamina* among pairwise tissue comparisons.**


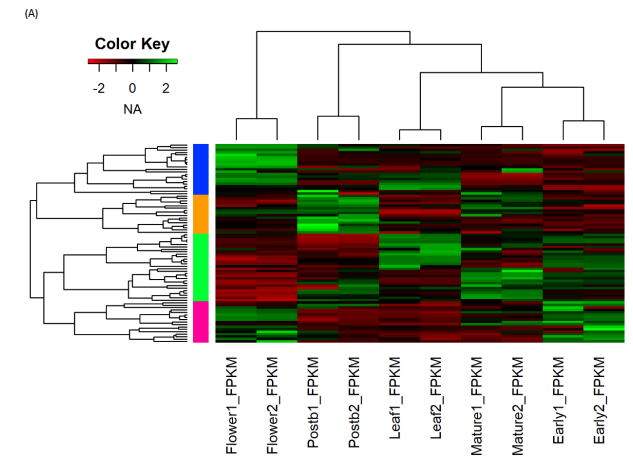


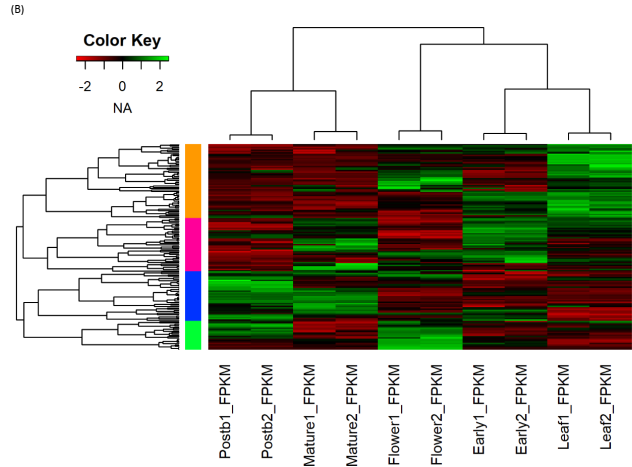

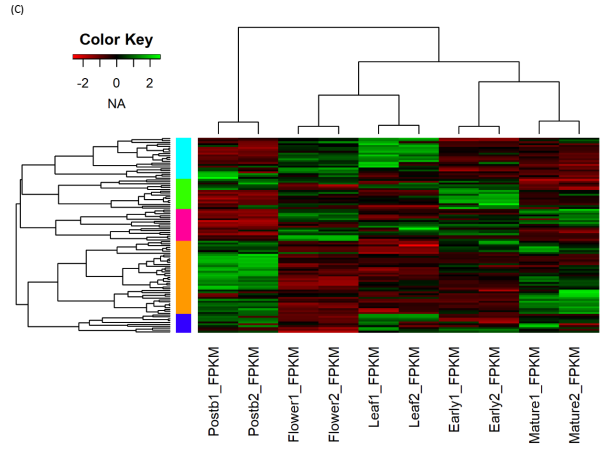


**Supplementary Figure S8. Hierarchical clustering of candidate genes encoding three enzymes postulated to be involved in the later steps of 2-methoxy-1,4-naphthoquinone (MNQ) biosynthesis.**


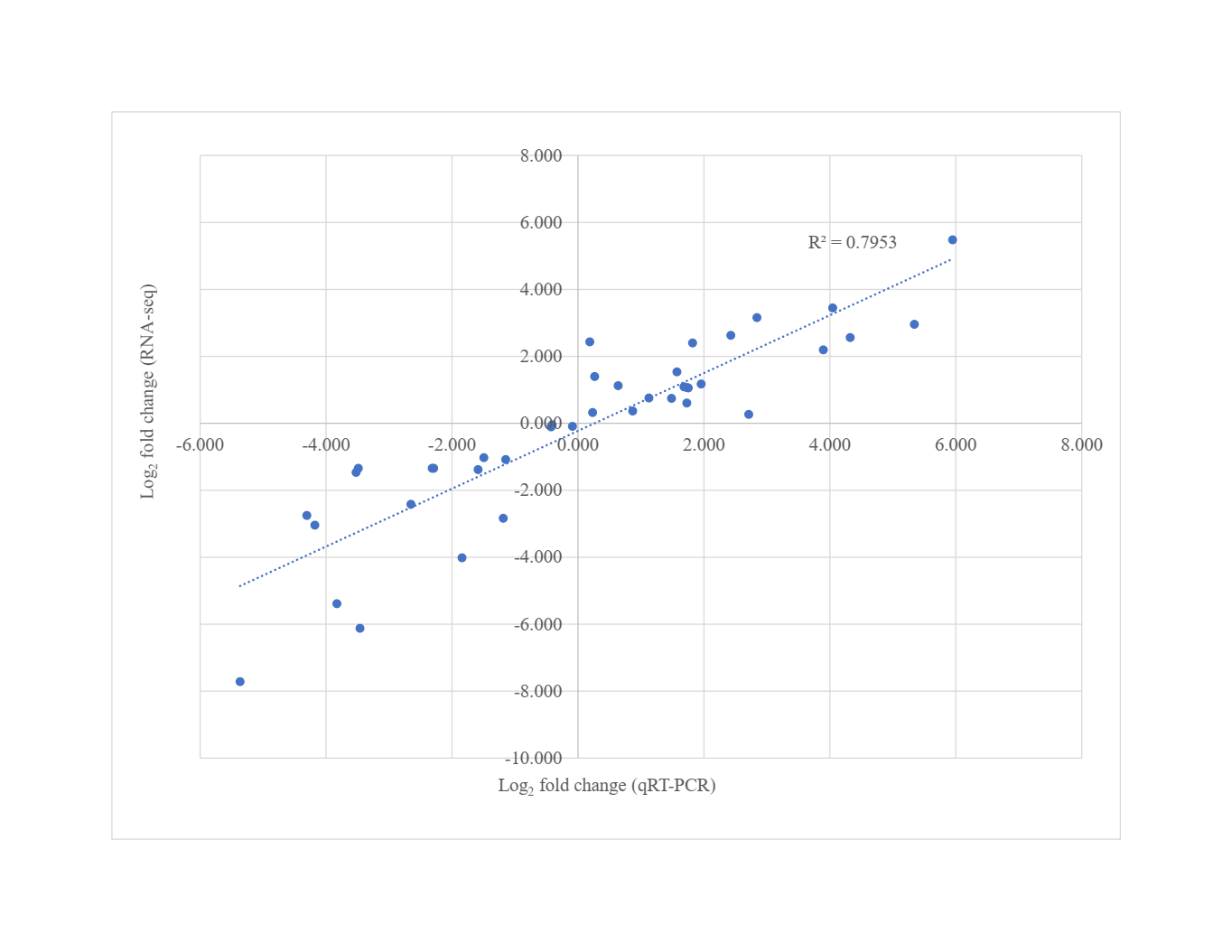


**Supplementary Figure S9. Linear regression analysis of gene expression data obtained from qRT-PCR and RNA-Seq.**
